# Adjuvant Effect of Mesoporous Silica SBA-15 of Different Morphologies on Antidiphtheria Immune Response

**DOI:** 10.1021/acsomega.5c03459

**Published:** 2025-06-19

**Authors:** Matheus C. R. Miranda, Carmen M. Nunes, Danilo W. Losito, Fernanda M. Rocha, Jéssica A. F. Pedro, Bruna C. Favoretto, Gabriel B. M. Teobaldo, LuÍs Carlos Cides da Silva, Jose L. S. Lopes, Cristiano L. P. Oliveira, Marcia C. A. Fantini, Orlando G. Ribeiro, Osvaldo A. Sant’anna, Tereza S. Martins

**Affiliations:** † Instituto de Ciências Ambientais, Químicas e Farmacêuticas, 28105 Universidade Federal de São Paulo, Diadema, São Paulo 09913-030, Brazil; ‡ Instituto de Física, 28133 Universidade de São Paulo, São Paulo 05508-090, Brazil; § Departamento de Física, Faculdade de Filosofia, Ciências e Letras de Ribeirão Preto, Universidade de São Paulo, Ribeirão Preto, São Paulo 14040-901, Brazil; ∥ 196591 Instituto Butantan, São Paulo 05503-900, Brazil

## Abstract

Ordered mesoporous silica SBA-15 improves the humoral response as a vaccine adjuvant because of its structural properties. Its morphology is dependent on synthesis conditions and can alter antigen encapsulation and immune response; all tested variants were safe and able to immunize against diphtheria. One of the additional advantages of SBA-15 is that its morphology can be modulated by adjusting the synthesis conditions like temperature, stirring speed, and solvent addition. In this study, SBA-15 was selected as a vaccine adjuvant in immunization against diphtheria by varying four modifications of the synthesis parameters for preparing SBA-15 particles with different morphologies. SEM analyses confirmed that different morphologies were obtained including rope-shaped aggregated rods (S1), filiform rods (S2), hexagons (S3), and nanospheres (S4). All synthesized SBA-15 samples presented an ordered mesoporous structure, with the characteristic reflections of a two-dimensional hexagonal structure and lattice parameter values with small differences (*a*(hkl) = ∼11 nm at 12), indicating that the silica mesostructure was preserved after incorporation from diphtheria anatoxin (dANA). The SAXS and NAI results indicate that mainly in samples S1 and S4, the dANA is encapsulated in the mesopores as well as in the SBA-15 macropores. Fluorescence analyses revealed the preservation of the aromatic microenvironment of tryptophan, similar to pure protein, except for sample S3, which showed a shift in emission wavelengths to 356 and 372 nm, indicating exposure of tryptophan to the more polar microenvironment. SRCD analyses confirmed the maintenance of dANA’s secondary structure in all samples. In the immunogenic assay, the S3dANA sample stood out, presenting a significantly higher primary immune response. However, the immunogenic responses increased and became equal in the secondary response without any variation between different silica morphologies. It is concluded that all SBA-15-based adjuvants with different morphologies are biocompatible and present a good immunogenic response when they are applied as vaccine adjuvants.

## Graphical abstract

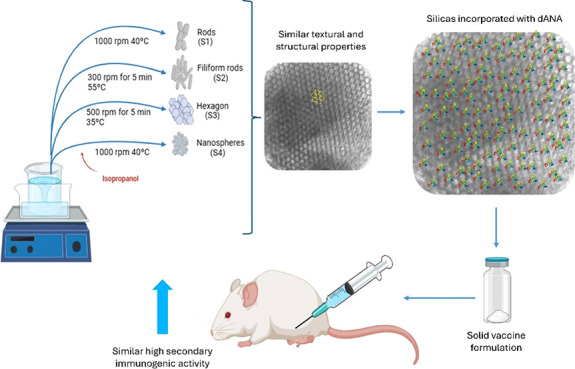

## 1 Introduction

Vaccines are one of the greatest inventions of all time, as they have made it possible to control and eradicate many types of infectious diseases such as smallpox and polio.^1^ In addition, the COVID-19 pandemic has stimulated the search for even more efficient vaccine formulations.^2–4^

Initially, vaccines were produced by using attenuated or inactivated pathogenic microorganisms as vaccine antigens. Effective vaccines, however, required the administration of large quantities of antigens.^5^ To address this issue, recombinant vaccines with purity and good safety have been developed, replacing some first-generation vaccines to immunize against various diseases. However, these vaccines exhibit low immunogenicity and do not induce a robust immune response due to the lack of exogenous components that activate the immune system, thus requiring an adjuvant to reinforce the immune response and increase their effectiveness.^6,7^ To increase the immunogenic activity of these vaccines, it was necessary to add other components known as adjuvants. They are substances that enhance vaccine efficacy by boosting the immune response when administered alongside vaccine antigens. These can range from synthetic compounds and small molecules to complex natural extracts and particulate materials.^8^

According to Facciolà et al.^1^ a good adjuvant must be safe, well-tolerated, and easy to produce; have good pharmaceutical characteristics (pH, osmolality, and endotoxin levels) and a durable shelf life; and be economically viable.

Vaccines that contain adjuvants in their formulation promote the maturation of a greater number of antigen-presenting cells (APCs), enhance the interaction between these APCs and T cells, and stimulate the production of a greater number and variety of polarizing T helper cytokines, multifunctional T cells, and specific antibodies. This leads to a broad and durable immune response as well as dose and antigen savings.^8,9^

Many types of adjuvants have already been successfully studied in vaccine formulations against different infectious diseases. Among these adjuvants are aluminum hydroxide, aluminum phosphate, aluminum hydroxyphosphate sulfate, and aluminum sulfate, which protect against diseases such as diphtheria, tetanus, pertussis, hepatitis A, and hepatitis B.^5^

Monophosphoryl lipid A (MPL) and QS-21 combined in a liposomal formulation are used as vaccine adjuvants against malaria. MPL combined with aluminum salt is used against human papillomavirus (HPV).^5^ Cytosine-phosphoguanine (CpG) is used against hepatitis B in adults. Squalene, Tween 80, and Span 85 are used to control the trivalent seasonal influenza vaccine.^5,10^

While there is a wealth of research in the field of vaccine adjuvants, the search for safer and more effective options is ongoing. Most FDA-approved adjuvants are based on aluminum salts, which, while effective, can lead to acute or chronic local inflammation with the formation of abscesses and nodules and induce hypersensitivity to the host’s tissues, potentially causing autoimmune arthritis. It is worth noting that the FDA recently approved liposome-based adjuvants for human use. However, these adjuvants are costly to produce and have low physical and chemical stability due to their fragile phospholipid membranes and peroxidation.^11^

For the reasons highlighted above, ordered mesoporous silica has been gaining prominence as an ideal candidate in the development of vaccine formulations. It offers the ability to incorporate proteins, drugs, and antigens, providing better stability and activity to the incorporated species and stimulating essential immunological memory.^12,13^ These characteristics are most likely attributed to the morphological and structural properties of SBA-15.^14^ SBA-15 has an ordered porous structure with a two-dimensional hexagonal shape corresponding to the p6mm space group, which gives it a high density of silanol groups that facilitate good interaction with vaccine proteins (antigens).^14,15^ The presence of silanol groups, a high surface area (up to 1000 m^2^ g^–1^), pore size (around 10 nm), pore volume (up to 1 cm^3^ g^–1^), and high thermal and chemical stability qualify SBA-15 as a material capable of adsorbing and controlling the release of antigens, as well as protecting the molecule to be released and degraded.^12,13,16,17^

The efficacy of SBA-15 as a vaccine adjuvant for different types of diseases has been demonstrated in several works in the literature. Immunogenicity tests have been carried out to prevent different types of antigens such as diphtheria, tetanus, hepatitis B, enzootic pneumonia, schistosomiasis, and bovine serum albumin (BSA-model antigen). All of the studies show that the SBA-15:antigen composites produced an immunogenic response almost three times higher than that of pure antigens^17^−.^18,19,20,21^

Notably, Rasmussen et al.^19^ obtained important results indicating that SBA-15 is more efficient as an adjuvant for diphtheria anatoxin (dANA) when compared to aluminum hydroxide. They showed higher antibody titers for oral and subcutaneous immunizations than the aluminum hydroxide adjuvant. Additionally, the SBA-15:dANA composite showed antibody titers more than twice as high as pure dANA, confirming that SBA-15 is an excellent adjuvant. Other results that corroborate these advantages of SBA-15 over aluminum hydroxide were obtained by Mercuri et al.^22^ who found that SBA-15 as an antigen carrier did not induce tissue damage, granuloma formation, or necrotic areas at the injection site, effects observed with aluminum hydroxide.

The work by Wang et al.^23^ demonstrated that modifying the textural properties of mesoporous silica, such as pore size, shape, and particle size, can induce different antigen release patterns and influence immunogenic activity when used as an adjuvant. However, no studies have investigated the impact of the SBA-15 morphology on its antigenic activity when employed as a vaccine adjuvant.

Inspired by the work of Lee et al.^24^ and Ding et al.^5^ who demonstrated the possibility of obtaining different morphologies of SBA-15 through simple adjustments to synthesis parameters (e.g., stirring speed and temperature) and noted a potential for incorporating vaccine antigens, the aim of this study was to evaluate the influence of different SBA-15 morphologies on dANA adsorption and immunogenic activity. This was done to verify whether these morphologies can influence antigen protection, release, and presentation to the immune system. Distinct morphologies can present varied characteristics, such as enhanced surface area, improved antigen accessibility, increased antigen protection, greater uptake by dendritic cells and macrophages, more precise control over antigen release, and enhanced exposure to the immune system. The influence of SBA-15 morphology on antigenic activity has not yet been addressed in the literature, as existing studies primarily evaluate the influence of characteristics such as SBA-15 particle size, pore size, and pore shape on antigenic activity. This novelty makes our work innovative and promising for developing SBA-15 vaccine adjuvants with optimized characteristics to improve immunogenic efficacy.

## 2 Materials and Methods

### 2.1 Sample Preparations

#### 2.1.1 SBA-15 Conventional Synthesis (S1)

SBA-15 was synthesized using 4 g of Pluronic P123 (poly­[ethylene oxide] – poly­[propylene oxide] – poly­[ethylene oxide], EO_20_PO_70_EO_20_; Sigma-Aldrich) dissolved in 30 mL of deionized water and 120 mL of a 2 mol L^–1^ HCl solution (Alphatec).^25^ The mixture was stirred at room temperature for 1 h until a homogeneous solution was achieved, after which 8.9 mL of tetraethyl orthosilicate (TEOS; Sigma-Aldrich) was added. The system was then heated and maintained at 40 °C for 24 h, followed by hydrothermal treatment in a Teflon-lined autoclave at 100 °C for 48 h. Subsequently, the solvent was removed by filtration and the resulting precipitate was washed with deionized water. To ensure the elimination of chloride ions, a nitrate test was performed by acidifying the medium with a 0.1 mol L^–1^ HNO_3_ solution (Sigma-Aldrich) and then adding a 0.1 mol L^–1^ AgNO_3_ solution (Synth). The structure-directing agent (Pluronic P123) was extracted using two steps: first, the SBA-15 was stirred in absolute ethanol for 2 h, followed by washing with ethanol. This procedure was repeated three times. Finally, the sample was calcined at 550 °C under an air atmosphere with a heating rate of 5 °C min^–1^ and maintained at this temperature for 3 h.

#### 2.1.2 Syntheses of S2 and S3

The samples designated as S2 and S3 were synthesized following the methodology outlined by Lee et al.^24^ who achieved different morphologies and particle sizes of SBA-15 by varying the synthesis temperature, stirring speed, and stirring time after adding TEOS. Initially, 4 g of Pluronic P123 was solubilized in 21 mL of deionized water and hydrochloric acid (HCl, 37%). The system was stirred magnetically in the silicone bath to solubilize the polymer for 3 h at 55 °C for sample S2 and 35 °C for sample S3. Subsequently, 9.0 mL of TEOS was added dropwise with stirring at 300 rpm for S2 and 500 rpm for S3. After adding TEOS, the mixture was stirred magnetically for 5 min. Finally, the mixture was left under static conditions for 24 h in a silicone bath at 55 °C (S2) and 35 °C (S3). The solution was then transferred from the stirring apparatus to a Teflon autoclave and heat-treated at 100 °C for 24 h. The generated solid was filtered and washed with deionized water. Following this process, solvent extraction using ethanol was performed to remove the copolymer, followed by filtration under reduced pressure and drying in a desiccator containing silica gel. The material was calcined at 550 °C under an air atmosphere at a rate of 5 °C min^–1^ and maintained at this temperature for 3 h.

#### 2.1.3 Synthesis of S4

Sample S4 was synthesized using the same method as that for sample S1, with the addition of isopropyl alcohol. In summary, 4.05 g of Pluronic P123 was dissolved in 15 mL of deionized water and 120 mL of 37% HCl. The system was magnetically stirred for 1 h to dissolve the polymer. Then, 7.5 mL of isopropyl alcohol and 4.5 mL of TEOS were separately added dropwise while stirring at 1000 rpm at 40 °C. After the addition of TEOS, the mixture was magnetically stirred for 24 min. The solution was transferred from the stirrer to a Teflon autoclave with a graphite outer body and heated at 100 °C for 24 h. The resulting solid was filtered and washed with deionized water. Subsequently, solvent extraction with ethanol was performed to remove the copolymer, followed by filtration under reduced pressure and drying in a desiccator containing silica gel. The material was calcined at 550 °C under an air atmosphere at a rate of 5 °C min^–1^ and maintained at this temperature for 3 h.

#### 2.1.4 Adsorption Process of Diphtheria Anatoxin (dANA) in Different Morphologies of SBA-15

The preparation of the immunogenic material (SBA:dANA) involved activating the SBA-15 at 190 °C for 2 h to remove the water molecules. Samples S1, S2, S3, and S4 were then added to a phosphate-buffered saline (pH ∼ 7.4) containing dANA. The resulting suspension was stirred for 24 h at 5 °C. The solvent was removed using the evaporation method, which consisted of placing the mixture in an oven at 35 °C for 2 days. The composites obtained were designed as S1dANA, S2dANA, S3dANA, and S4dANA. The composite mass ratio utilized was 10SBA-15:1dANA:8.7PBS salts (NaCl, KCl, KH_2_PO_4_, Na_2_HPO_4_) representing the final nominal concentration of the composites.

### 2.2 Samples Characterizations

Small angle X-ray scattering (SAXS) measurements were performed using the NANOSTAR instrument (Bruker, MA, USA) equipped with a microfocus Genix3D X-ray source, Fox3D focusing system, and two sets of scatterless slits, all provided by Xenocs. The wavelength was fixed at Cu–Kα (λ = 0.15418 nm), and data acquisition was carried out for 900 s with the collimation set to ultrahigh resolution (beam size of 0.3 × 0.3 mm). The 2D scattering image was collected on a Dectris-Pilatus300k pixel detector. The detector is integrated inside the vacuum system, allowing a beam-stopperless setup. The obtained 2D images were integrated using a program package.^26^ The sample-detector distance was set as 65 cm (0.2 ≤ *q* ≤ 3.5 nm^–1^).^19^ The same samples were analyzed by USAXS measurements at the Xeuss 2.0 system at the EMUSAXS center.^27^ This system is also equipped with a microfocus source Genix3D (Cu–Kα, λ = 0.15418 nm), Fox3D mirrors, and two sets of scatterless slits. The acquisition times were 900 s with the collimation set to high resolution (beam size of 0.7 × 0.7 mm). The 2D scattering images were also collected on a Dectris-Pilatus300k pixel detector and the images were integrated using the program package FIT2D. In both cases, the data treatment was done with the program SUPERSAXS;^28^ background subtraction and error estimation were done with this program.

Nitrogen adsorption–desorption isotherms (NAI) data were collected using a Micromeritics ASAP 2020 instrument. Prior to analysis, the samples were subjected to degassing (<10 μm Hg) at 40 °C. The nitrogen adsorption analysis was conducted at a temperature of −196 °C. The obtained isotherms were analyzed using the BET (Brunauer–Emmett–Teller) method to determine the specific surface area and the BJH (Barrett–Joyner–Halenda) method with the KJS (Krug–Jaroniec–Sayari) thickness equation for pore size distribution values.^19^

Scanning electron microscopy (SEM) images and energy dispersive X-ray spectroscopy (EDS) spectra were acquired using a JEOL JSM-6610 Microscope (JEOL, Tokyo, Japan) operating at an analysis voltage of 3 kV. The SEM images were obtained at magnifications of 1000 and 10 000× (20 000 and 30 000× for sample S4). Prior to analysis, the powdered samples were deposited onto a carbon tape and subsequently metallized with gold for enhanced conductivity and imaging quality.^29^

Thermogravimetric analysis (TG) and differential scanning calorimetry (DSC) measurements were performed by using a TA Instruments Discovery SDT 650 thermobalance. The analysis was conducted over a 25 to 1000 °C temperature range with a heating rate of 10 °C min^–1^ under a dynamic air atmosphere with a flow rate of 50 mL min^–1^. The samples were placed in 90 μL alumina crucibles.

Fourier transform infrared (FTIR) spectra were recorded in the range of 4000–400 cm^–1^, using an Agilent Cary 630 FTIR spectrometer. IR measurements were performed in attenuated total reflection (ATR) mode with 1024 scans^.^ .^29^

Synchrotron radiation circular dichroism (SRCD) spectroscopy measurements were collected on the AU-CD beamline of the ASTRID2 synchrotron at Aarhus University (Denmark) by using the Periscope system, where the light beam is incident vertically on the sample. After dispersing the particles in aqueous solution, measurements were carried out with diphtheria toxin in solution and incorporated into silicas with different morphologies (S1, S2, S3, and S4). The SRCD spectra were collected from 280 to 170 nm at intervals of 1 nm and 2.0 s of dwell time, at 25 °C, using a cylindrical Suprasil quartz cuvette with an optical path length of 99.2 μm. The final spectra were processed using the CDToolX software^30^ by averaging the six individual scans, subtracting the SRCD spectrum of the respective silica in buffer, and zeroing between 265 and 270 nm.^30^ The composite spectra were normalized to the absorption intensity of dANA in solution at 222 nm.

Fluorescence measurements were carried out using a Fluorolog 3 FL3-22 Spectrophotometer (HORIBA Jobin Yvon, Kyoto, Japan). dANA and SBA-15 samples incorporated with dANA were placed in the sample holder for solids, employing a front-face configuration. An excitation wavelength of 280 nm was used, and the analysis range spanned from 290 to 500 nm.^31^

### 2.3 Biocompatibility Analysis

#### 2.3.1 Hemolytic Activity

The protocol used was a modification of Onuma et al.^32^ wherein this experiment, defibrinated sheep’s blood red blood cells (NEW PROV, Batch number: 77719, Paraná, Brazil) were used to conduct a preliminary toxicity study before *in vivo* application. 100 μL of a dispersion containing 100 μg SBA-15 (different morphologies) incorporated with 10 μg dANA was incubated in PBS and 100 μL of erythrocyte solution at 37 °C for 1 h. The tubes were centrifuged at 3000 rpm for 3 min, and 50 μL aliquots of the supernatant were pipetted into 96-well microplates. The absorbance at 540 nm was determined by using a microplate reader (Biotek, Model: Power Wave 22). The value of 100% hemolysis was determined using 50 μL of PBS buffer with 100 μL of 1% (v/v) Triton X-100, while the value of 0% hemolysis was obtained using 10 μL of PBS buffer.^9^

### 2.4 *In Vivo* Immunogenic Analysis

#### 2.4.1 Mice

We used 3-month-old HIII male and female mice raised in the animal facilities of the Immunogenetics Laboratory at the Butantan Institute (São Paulo, Brazil), and the experiments were conducted according to the Butantan Institute Animal Ethics Committee (#9894070224).

#### 2.4.2 Preparation of Immunogenic Complexes (SBA-15:dANA) and Mouse Immunization

The immunogenic complex (SBA-15:dANA) was prepared in a PBS buffer solution using a mass ratio of 1:10 (SBA-15:dANA). The system was left to rest and stabilize for 24 h at 4 °C. Subsequently, HIII mice were subcutaneously immunized with the immunogenic complexes, each dose containing 10 μg of dANA (diphtheria anatoxin) incorporated into different SBA-15 preparations, designated as S1, S2, S3, and S4. The HIII mice were inoculated with SBA-15:dANA complexes on days 0 and 40.

#### 2.4.3 Antibody Titration

To determine anti-dANA antibody titers, we used the ELISA test on serum samples collected 30 days after each immunization dose.^33^ Briefly, the microplates were coated with 1 μg/well of dANA in 0.1 mol L^–1^ NaHCO_3_ buffer and incubated at 4 °C overnight. After a wash cycle using PBS containing 0.05% Tween 20 [PBS-T], the plates were blocked for 1 h at 37 °C with 0.01% gelatin-PBS-T. Following another wash cycle, serial dilutions of the serum samples were added, and the plates were incubated for 1 h at 37 °C. After an additional wash cycle, peroxidase-labeled mouse IgG (KPL, USA, 1:7,500) was added. After washing again, the reaction was developed at room temperature with 0.5 mg mL^–1^ *o*-phenylenediamine dihydrochloride (Sigma-Aldrich) and 0.03% H_2_O_2_ (Merck), then stopped with 0.2 N H_2_SO_4_. The absorbance was measured at 450 nm, and antibody titers were calculated as the reciprocal dilution of the serum that produced an absorbance value two standard deviations above the mean obtained from a control pool of normal sera. The results were expressed as [log^2^[xmean ± SEM]].^18^

## 3 Results and Discussions

### 3.1 Samples Characterizations

#### 3.1.1 Study of the Different Morphologies of SBA-15 Silicas

The morphology of SBA-15 particles was analyzed by SEM to confirm the formation of different SBA-15 particle morphologies resulting from changes in the synthesis parameters. The SEM micrographs (Figure 1) show that the particles of sample S1, synthesized at 40 °C and stirred at 1000 rpm (conventional synthesis), have a homogeneous rod-shaped morphology aggregated in the form of a rope, with an average length of 1.15 ± 0.13 μm and an average width of 0.47 ± 0.08 μm^31^. Samples S2 and S3 were synthesized using the methods proposed by Lee et al.^24^ Sample S2, synthesized at 55 °C and stirred at 300 rpm, displayed a homogeneous morphology of filiform rods with an average length of 1.34 ± 0.21 μm and an average width of 0.25 ± 0.04 μm. Sample S3, synthesized at 35 °C and stirred at 500 rpm, exhibited a homogeneous hexagonal morphology with an average length of 1.338 ± 0.318 μm and an average width of 1.01 ± 0.15 μm. Sample S4 was synthesized using the same methodology as for sample S1, with the exception of the addition of isopropyl alcohol. The SBA-15 particles in this sample showed a morphology of nanospheres with an average size of 272.4 ± 0.13 nm.

**Figure 1:**
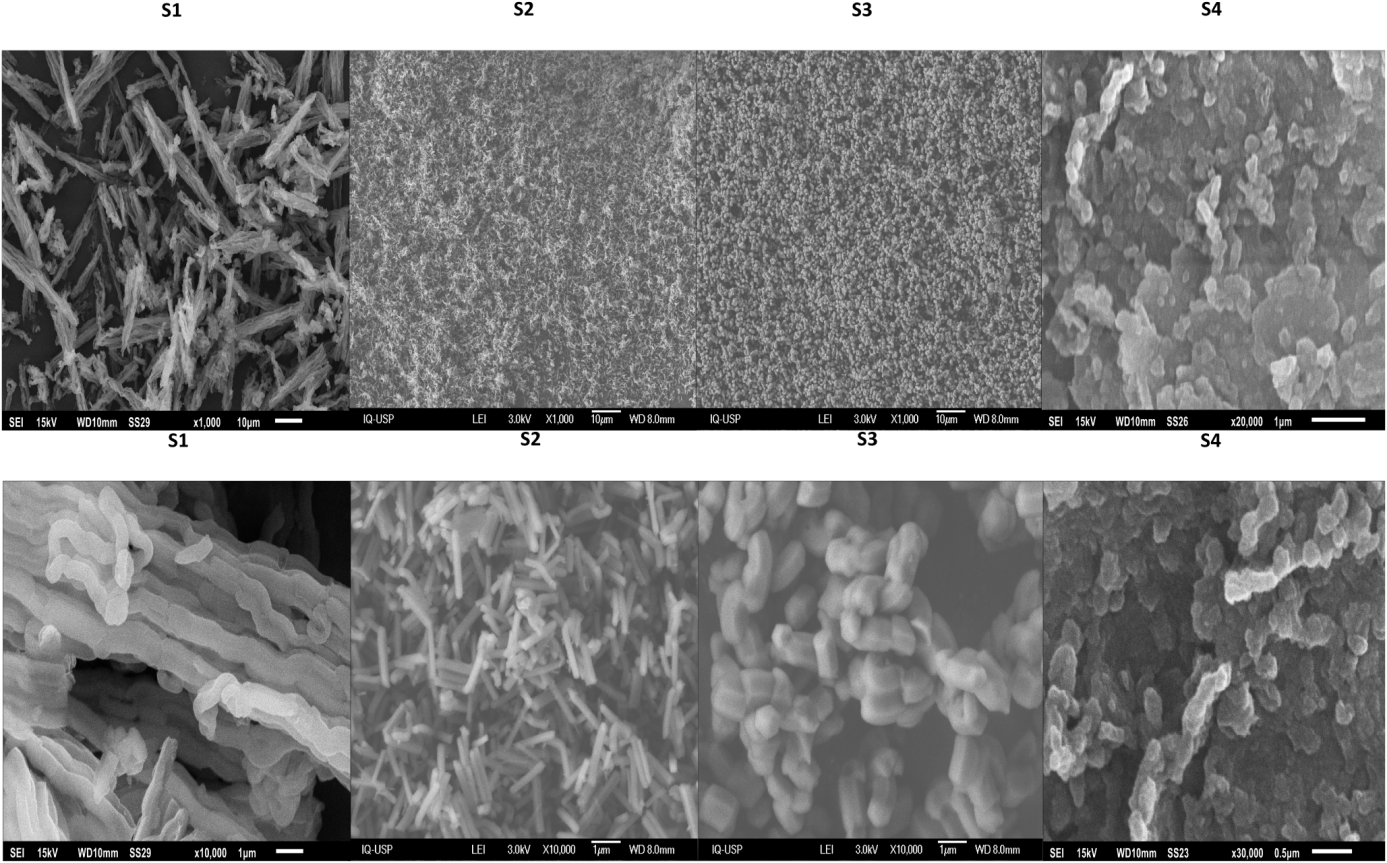
SEM micrographs of S1, S2, S3, and S4 samples: low- and high-magnification images revealing the different morphologies of SBA-15 samples affected by synthesis parameters. The upper part scales: S1, S2, and S3 are 10 μm and S4 is 1 μm. Lower part scales: S1, S2, and S3 are 1 μm and S4 is 0.5 μm.

These results demonstrate that nucleation synthesis conditions, such as the temperature and stirring speed, are crucial factors for the systematic morphological control of SBA-15 particles. According to Lee et al.^24^ higher temperatures accelerate the rate of hydrolysis and condensation of silica precursors, shortening the time for the growth of nanodomains into large particles. Additionally, slower stirring rates may provide fewer opportunities for particle growth, while higher stirring rates may promote greater opportunities for particle growth.^24^

Sample S3 (35 °C, 500 rpm) had a hexagonal shape similar in length to the other samples but with a width four times greater. Another result corroborating Lee et al.^24^ is that stirring speeds above 500 rpm favored the formation of rod and hexagonal morphologies, while speeds above 300 rpm favored thinner rod morphologies.

The reduced size of the nanosphere in sample S4 is likely due to the slower hydrolysis of TEOS caused by the addition of isopropyl alcohol. This effect was easily observed during the synthesis due to the delayed condensation of silica, as isopropyl alcohol acts as a solvent and can interfere with the chemical reactions involved in the formation of silica structures.^34,35^

Figure 2 shows the SAXS curves of the pure SBA-15 samples (S1, S2, S3, and S4), where all samples exhibit five diffraction reflections, corresponding to the Miller indices (hkl): (100), (110), (200), (210), and (300). These reflections indicate that the analyzed SBA-15 samples (Table 1) have a mesoporous arrangement with a two-dimensional hexagonal structure, corresponding to the p6mm space group, as reported in the literature.^13,15,17,31,36^ Additionally, the lattice parameters (Table 1) show that samples S1 and S2 have larger lattice parameters of approximately 12 nm, while samples S3 and S4 have lattice parameters of approximately 11 nm. These results suggest that despite the significant differences in morphologies and particle sizes generated by the different synthesis parameter modifications, the lattice parameter values are close. These findings are consistent with the work of Lee et al.^24^

**Figure 2:**
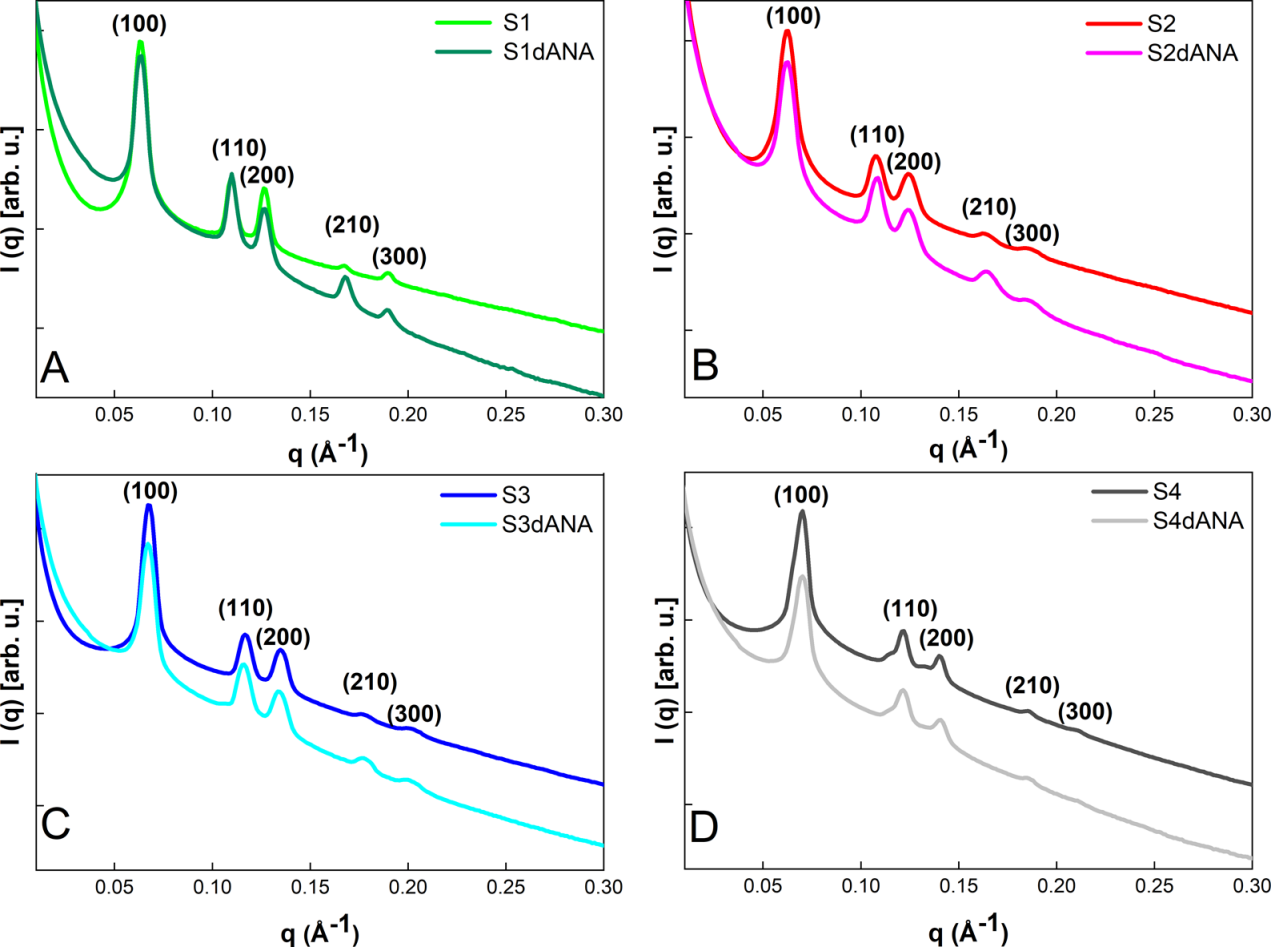
SAXS curves of the SBA-15 and SBA-15:dANA samples: A) S1 and S1dANA; B) S2 and S2dANA; C) S3 and S3dANA; and D) S4 and S4dANA.

**Table 1:** Structural Properties Determined by SAXS Curves of Samples S1, S2, S3, S4, S1dANA, S2dANA, S3dANA, and S4dANA^a^

	d_(hkl)_ (nm)					a_(hkl)_ (nm)				
Samples	(100)	(110)	(200)	(210)	(300)	(100)	(110)	(200)	(210)	(300)
S1	10.0	5.8	5.0	3.8	3.3	11.5	11.5	11.5	11.5	11.5
S2	10.1	5.9	5.1	3.8	3.4	11.7	11.7	11.7	11.7	11.6
S3	9.4	5.4	4.7	3.5	3.1	10.8	10.8	10.8	10.9	10.8
S4	9.0	5.2	4.5	3.4	3.0	10.4	10.4	10.4	10.4	10.4
S1dANA	10.0	5.7	5.0	3.7	3.3	11.5	11.4	11.5	10.4	11.5
S2dANA	10.1	5.8	5.0	3.8	3.4	11.6	11.6	11.6	11.7	11.7
S3dANA	9.4	5.4	4.7	3.5	3.1	10.9	10.9	10.8	10.8	10.8
S4dANA	9.0	5.2	4.4	3.4	3.0	10.4	10.3	10.3	10.4	10.4

a *a*(hkl) = lattice parameter; *d*(hkl) = interplanar distance; The error of the lattice parameter is 2%.

After dANA was incorporated into the different SBA-15 morphologies, the samples were analyzed again by SAXS (Figure 2). The results show structural parameters, including interplanar spacing (*d*_(hkl)_) and lattice parameter (*a*_(hkl)_), quite close to those of the pristine samples, suggesting that the different synthesis processes employed, and the dANA incorporation process did not induce significant changes in the mesoporous structure (Table 1).

In the region of extremely small scattering angles (*q* < 0.05 Å^–1^) (Figure 2), all the incorporated samples showed greater scattering intensity than the pristine samples. Samples S4dANA and S1dANA exhibited a larger difference in scattering intensity compared with pristine samples S1 and S4. In contrast, samples S2 and S3 showed much less significant differences in scattering intensity in the *q* range less than 0.05 Å^–1^. This greater dispersion in samples S4dANA and S1dANA can be attributed to the presence of protein aggregates inside the SBA-15 macropores, suggesting that these samples have a superior amount of dANA aggregates inside their macropores.^19,31^

Another noteworthy observation in the SAXS curves in Figure 2 is the reduction in the scattering intensity of the diffraction peaks (100), (110), and (200) in the samples incorporated with dANA compared to the pure SBA-15. This reduction can be attributed to the contrast in electron density between the mesopores and the pore walls of the silica matrix, which is diminished by the presence of dANA or PBS constituents within the silica mesopores.^31,33^

In order to obtain a more detailed characterization of the structure, we applied an advanced modeling procedure to the SAXS data. This model is based on the work of Sundblom et al.^37^ and assumes that the system consists of core–shell cylinders packed in a hexagonal arrangement. In this model, the scattering intensity is described by Miranda et al.:^31^

1 $$I\left(q\right)=S_{C1}P_{rod}\left(q\right){\langle F}_{CS}{\left(q\right)}^{2}\rangle (1+\beta \left(q\right)\left[\langle Z\left(q)\right\rangle -1\right]G\left(q\right))+S_{C2}I{\left(q\right)}_{chain}+S_{Cextra}I{\left(q\right)}_{extra}+Back$$

where *P*_rod_(*q*) is the intensity form factor of an infinitely thin rod with length *L*, *F*_CS_(*q*) is the amplitude form factor of a core–shell cylinder with inner radius *R*_in_, outer radius *R*_out_, relative polydispersity σ_R_, and the ratio between outer shell and inner core contrasts given by Δρ_R_ (Δρ_R_=Δρ_out_/Δρ_in_). The structure factor is weighted by the parameter *c* with lattice parameter *a*, domain size *D*, and a disorder factor for the cylinders’ packing σ_dis_. The polymer chains used during synthesis give rise to microporosities that are present even for calcination and can be described by Gaussian chains with a radius of gyration RG_chain_ and is included in the factor *I*(q)_chain_. After several tests, we concluded that the SAXS data from samples with incorporated material exhibit additional contributions to the scattering intensity, which were described as ellipsoids of rotation with radius *R*_ext_ and anisotropy ε_ext_. *S*_C1_, *S*_C2_, and *S*_Cext_ are the overall scale factor, the scale factor of the Gaussian chain, and the scale factor of the extra factor, respectively. Finally, a constant background, *back*, is also added to the model. Mathematical details are found in the.^31,37^

The fits for the experimental data are listed in Figure 3. As shown in this figure, excellent fits were obtained using the proposed models. The corresponding model parameters are shown in Tables 2 and 3. Small variations in the lattice parameter *a* were observed depending on the synthesis route, ranging from 10.3 to 11.7 nm. Sample S4 exhibits two phases (double peaks), and from the modeling we obtained the parameters for each phase (S4–C1 and S4–C2). The fits for each phase are shown in Figure S2. The mesoporous inner radius varies from ∼4.3 to ∼4.9 nm. The chain radius of gyration was set to ∼1.4 nm and the cylinder length to ∼200 nm, since they were unstable for optimization (Table S1). Interestingly, for the samples with incorporated material, it was necessary to add an extra term. For samples S1, S2, and S3, the additional term was given by large oblate ellipsoids. For sample S4, the additional term was given by long prolate ellipsoids. Note that this additional term is tentative to describe the incorporated material in the system and might provide some indications about it.

**Figure 3:**
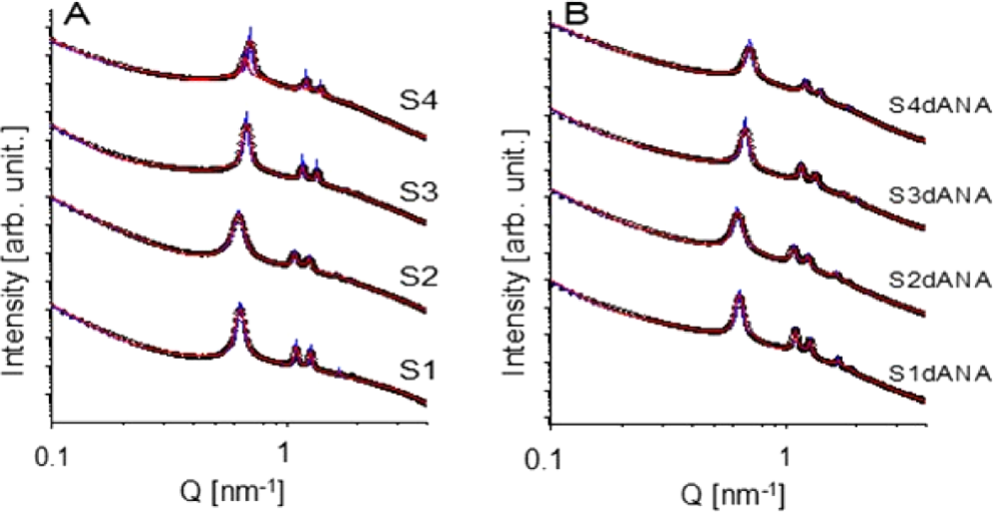
Advanced modeling was performed for the samples investigated. Symbols: experimental data. Red solid lines: smeared data. Blue solid lines: desmeared data. For sample S4 and S4dANA the results for the two phases are shown.

**Table 2:** Obtained Model Parameters for the Pure Samples

Parameters	S1	S2	S3	S4-C1	S4-C2
SC_CHAIN_	0.174 ± 0.001	0.321 ± 0.005	0.33 ± 0.01	0.391 ± 0.007	0.362 ± 0.006
*C*	8.4 ± 0.9	14.5 ± 0.6	15.8 ± 0.7	7.0 ± 0.4	2.2 ± 0.2
*A* (Å)	114.60 ± 0.04	116.40 ± 0.07	107.70 ± 0.06	103.80 ± 0.06	109.4 ± 0.2
*D* (×10^3^ Å)	5.4 ± 0.2	2.78 ± 0.08	8.7 ± 0.3	9.4 ± 0.4	3.5 ± 0.2
Σ_DIS_	0.047 ± 0.002	0.085 ± 0.002	0.101 ± 0.002	0.105 ± 0.002	0.096 ± 0.002
*R* (Å)	40.0 ± 0.3	49.0 ± 0.3	45.1 ± 0.3	43.7 ± 0.2	44.2 ± 0.2
Σ_REL_	0.061 ± 0.002	0.118 ± 0.002	0.116 ± 0.002	0.116 ± 0.002	0.126 ± 0.002
*R*_OUT_ (Å)	72.3 ± 0.3	67.7 ± 0.3	61.0 ± 0.2	62.0 ± 0.3	61.2 ± 0.3
RG_CHAIN_ (Å)	∼14	∼14	∼14	∼14	∼14
SC_EXT_	––	––	––	–	–
*R*_EXT_ (Å)	–	–	–	–	–
*E*_\EXT_	–	–	–	–	–

**Table 3:** Obtained Model Parameters for the Incorporated Samples

Parameters	S1dANA	S2dANA	S3dANA	S4dANA-C1	S4dANA-C2
SC_chain_	0.00427 ± 0.00007	0.0118 ± 0.0005	0.0120 ± 0.0005	0.0251 ± 0.004	0.0251 ± 0.005
*C*	6.7 ± 0.8	14.1 ± 0.7	16.2 ± 0.9	6.7 ± 0.3	6.1 ± 0.5
*a* (Å)	114.2 ± 0.05	116.00 ± 0.07	107.9 ± 0.05	103.1 ± 0.08	105.9 ± 0.3
*D* (×10^3^ Å)	4.0 ± 0.2	1.90 ± 0.06	2.63 ± 0.09	2.5 ± 0.1	2.9 ± 0.4
σ_dis_	0.052 ± 0.002	0.066 ± 0.002	0.067 ± 0.003	0.082 ± 0.002	0.115 ± 0.007
*R* (Å)	46.7 ± 0.3	48.9 ± 0.3	44.6 ± 0.3	42.5 ± 0.1	42.7±0.2
σ_rel_	0.075 ± 0.003	0.125 ± 0.003	0.147 ± 0.003	0.111 ± 0.002	0.121 ± 0.004
*R*_out_ (Å)	72.3 ± 0.3	67.7 ± 0.3	61.0 ± 0.2	62.1 ± 0.3	62.1 ± 0.3
RG_chain_ (Å)	∼14	∼14	∼14	∼14	∼14
SC_ext_	9 ± 3	34 ± 3	4.7 ± 0.5	1.2 ± 0.2	1.1 ± 0.2
*R*_ext_ (Å)	305 ± 55	489 ± 22	177 ± 9	21.6 ± 0.2	21.3 ±0 .2
ε_\ext_	0.031 ± 0.005	0.037 ± 0.002	0.087 ± 0.007	7.9 ± 0.2	9.0 ± 0.2

To improve the understanding of the models, it is important to highlight that the radius of gyration of the polymer chains (RG_chain_) indicates the presence of residual micropores formed by the Pluronic P123 used as a pore-directing agent during synthesis. These micropores may serve as potential adsorption sites for dANA. The values obtained for the internal and external radii of the cylindrical pores (*R*_in_ and *R*_out_) suggest that, despite variations in synthesis conditions (e.g., temperature, stirring, and ethanol addition), the mesopore diameters remained largely unchanged across different SBA-15 morphologies, both the pure SBA-15 samples and those loaded with dANA (SBA-15:dANA samples). A particularly insightful outcome from the modeling was the need to include an additional scattering term, represented as ellipsoids, to accurately describe the SAXS/USAXS profile of the dANA-loaded samples. As shown in Tables 2 and 3, the S1dANA and S2dANA samples exhibited oblate ellipsoids with low eccentricity (ε_ext_ <0.1), suggesting that the dANA clusters were organized in flattened configurations near the mesopore entrances. In contrast, the S4dANA sample displayed highly elongated prolate ellipsoids (ε_ext_ ≈ 8), indicating the formation of fibrillar or linear aggregates. These are likely accommodated within the macropores formed by the aggregation of nanospheres, an observation consistent with the high degree of particle aggregation and nanometric size confirmed by electron microscopy. These findings underscore that the morphology of SBA-15 plays a critical role not only in the spatial distribution of dANA within the pores but also in its supramolecular organization (e.g., particle aggregation). This organization could, in turn, affect both the release profile of dANA and its recognition by immune cells.

The USAXS data and modeling are presented in the Supporting Information to provide an overall indication of the aggregate sizes and shapes. The IFT method was applied using the GNOM program.^38^ The scattering curves, model fits, and obtained *p*(*r*) curves are shown in Figure S2. The overall behavior of the *p*(*r*) curves suggests that the agglomerates have globular shapes. To obtain a pictorial representation of the agglomerate shapes, the DAMMIN software package was used.^39^ In this modeling approach, an initial spherical search space is filled with spherical subunits, and the subset that provides the best fit of the scattering data is determined. For each sample, 10 independent runs were performed, and the most probable shape was obtained using the program.^40^ The resulting models are shown in Figure S3. Even though the agglomerates might have various sizes and shapes, this approach provides an average approximation.

Figure 4A,B shows that nitrogen adsorption isotherms (NAI) for SBA-15 samples are similar to those ones reported by Thommes et al.^41^ The isotherm of SBA-15 samples showed a hysteresis loop with sharp adsorption and desorption branches. The sharpness of the branched adsorption is indicative of a narrow pore size distribution (PSD), as shown in Figure 4C,D. The branched adsorption was located at relative pressures in the 0.65–0.8 range, a high relative pressure, similar to that of good-quality mesoporous materials. The textural and structural properties of the pure SBA-15 and SBA-15:dANA samples are shown in Table 4, and these parameters were similar to the ordered mesoporous materials with hexagonal structures, in good agreement with those reported for SBA-15.

**Figure 4:**
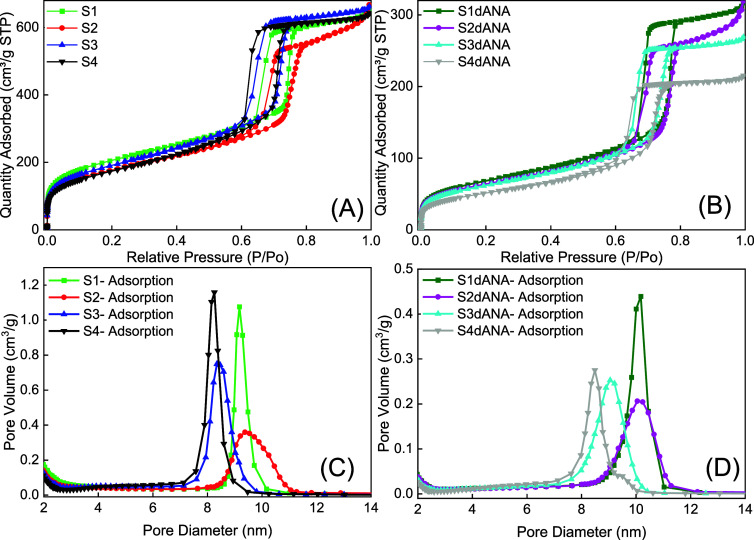
Nitrogen adsorption–desorption isotherms (A,B) and pore size distributions (C,D) of the pure SBA-15 and SBA-15:dANA samples, respectively.

**Table 4:** Results Obtained from NAI Analysis

Samples	BET surface area (m^2^ g^–1^)	Single point pore volume (cm^3^ g^–1^)
S1	739	1.01
S2	635	1.03
S3	686	1.02
S4	625	0.99
S1dANA	245	0.49
S2dANA	229	0.49
S3dANA	223	0.42
S4dANA	184	0.33

The NAI analyses of the pure SBA-15 samples (Figure 4A,C) show that samples S1 (739 m^2^ g^–1^) and S3 (686 m^2^ g^–1^) had higher mesoporous specific surface areas (determined by the BET method), while samples S2 (635 m^2^ g^–1^) and S4 (625 m^2^ g^–1^) had lower surface areas but close values, as can be seen in Figure 4 and Table 4. However, all pristine SBA-15 samples had similar pore volumes (around 1 cm^3^ g^–1^). The isotherms were identified as type IV (Figure 4A,B) and the hysteresis loop as type H1, according to the IUPAC classification for mesoporous materials.^41^

The pore distribution results (Figure 4C,D) showed that samples S2 (9.5 nm) and S1 (9.2 nm) had the largest pore diameters, while samples S4 (8.2 nm) and S3 (8.4 nm) had the smallest. Notably, the difference between the pore diameters and the lattice parameters (SAXS) provides an approximate value of the mesopore wall thickness: 2.3 nm for sample S1, 2.2 nm for sample S2, 2.4 nm for sample S3, and 2.1 nm for sample S4.

The incorporation of dANA into various silica matrices (S1, S2, S3, and S4) led to a consistent decrease in their pore volumes. Based on the pore volume data (Table 4), the occupancy rates of SBA-15 mesopores were 49% for S1, 48% for S2, 41% for S3, and 33% for S4 samples.^42^ The same trend was noted in the specific surface area values (Table 4), which were lower than those of the respective pristine silica samples. The decrease in the values of pore volume and specific surface area in the composites (S1dANA, S2dANA, S3dANA, and S4dANA) indicates the incorporation of dANA and constituents of the PBS buffer within the mesopores of the silica matrices.

Miranda et al.^31^ incorporated BSA into SBA-15 using phosphate-buffered saline (PBS) as the incorporation solvent, similar to this study. To test the behavior of PBS components on adsorption onto SBA-15, they incorporated only PBS into SBA-15, obtaining mesopore volume values (evaporated: 227 m^2^ g^–1^; freeze-dried: 272 m^2^ g^–1^) similar to those in this study for samples incorporated with dANA. These results indicate competition between the species present (salts and protein), mainly due to the smaller metal ions favoring migration to the mesopores. Consequently, BSA^31^ or dANA is also retained in the macropores. These findings are consistent with the results reported by Rasmussen et al.^19^ who also obtained similar values with SBA-15 incorporated with dANA. Additionally, Rasmussen et al.^19^ calculated the structural size of dANA, around 10 nm long and ∼3–4 nm wide, to enter SBA-15 mesopores.

The SAXS and NAI results of SBA-15:dANA samples suggest that a fraction of dANA is encapsulated within the mesopores of the SBA-15 along with PBS components, while the remainder is likely within the macropores of SBA-15. This effect is more pronounced in the S4dANA sample, which shows greater amounts of protein aggregates inside the macropores (from SAXS analysis), a smaller surface area, a smaller mesopore size, and a lower mesopore occupancy rate. Samples S1 and S2 showed a higher mesopore occupancy rate and fewer protein aggregates compared to S4, indicating a greater possibility of mesopore occupancy with the dANA and the PBS components.^19^

The results of this study indicate that dANA (protein aggregates) can also be incorporated into the macroporosity of SBA-15.^20,21,36^

Figure 5 and Table 5 present the TG and DSC analyses of pure SBA-15 and SBA-15 incorporated with dANA (SBA-15:dANA composites). In the TG analysis (25 to 1000 °C), two weight loss events are observed for pristine SBA-15 samples (S1, S2, S3, and S4), while three events are observed for SBA-15:dANA composites (S1dANA, S2dANA, S3dANA, and S4dANA). For both pure SBA-15 samples and SBA-15:dANA composites, the first weight loss event (I) occurs from 25 to 180 °C, resulting in a weight loss ranging from 2.8% (S1dANA) to 15.6% (S2) (Table 5). This loss corresponds to the elimination of adsorbed water and is accompanied by an endothermic peak in the DSC curves (Figure 5).

**Figure 5:**
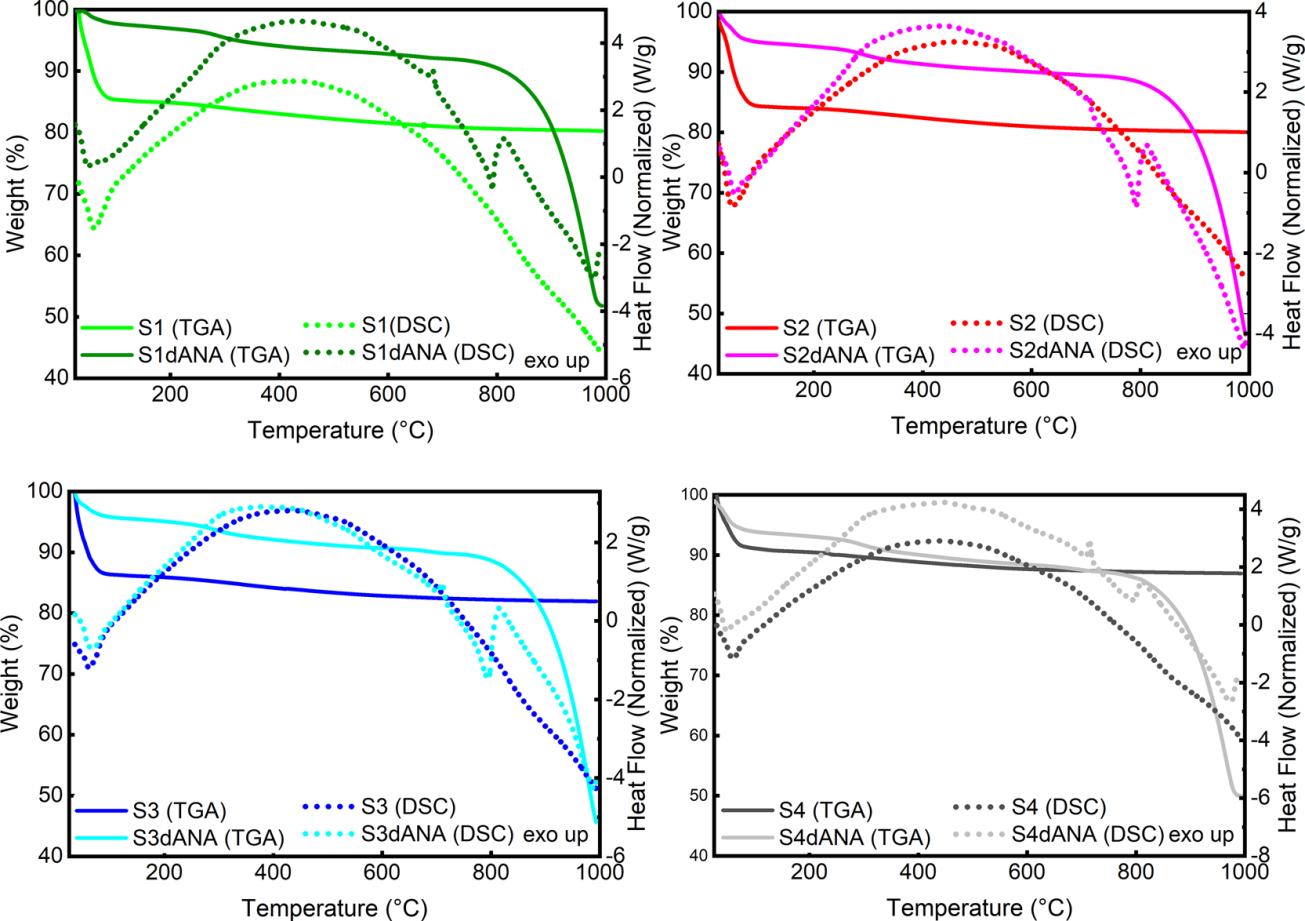
TG and DSC curves of pure SBA-15 samples (S1, S2, S3, and S4), and SBA-15:dANA composites (S1dANA, S2dANA, S3dANA and S4dANA).

**Table 5:** Results Obtained from TG and DSC Curves of Pure SBA-15 Samples (S1, S2, S3, and S4) and S1dANA, S2dANA, S3dANA, and S4dANA Composites^a^

Samples	1st step (25–180 °C)ΔW (T_onset_ °C)	2nd step (180–1000 °C)^1^ (180–650 °C)^2^ΔW (T_onset_ °C)	3rd step (650–1000 °C)^2^ΔW (T_onset_ °C)	Melting point Temperature (°C)
S1	14.2 (34)	4.1 (262)	-	-
S2	15.6 (40)	4.0 (270)	-	-
S3	13.0 (38)	4.1 (284)	-	-
S4	8.7 (32)	3.7 (246)		
S1dANA	2.8 (43)	5.0 (260)	42.4 (896)	681.1
S2dANA	5.1 (31)	5.0 (260)	43.0 (910)	709.9
S3dANA	5.0 (37)	5.4 (261)	43.0 (907)	710.8
S4dANA	6.3 (23)	4.9 (264)	40.8 (897)	714.9

a *T* = Temperature, Δ*W* = weight loss, 1SBA-15, 2SBA-15:dANA composites.

The second weight-loss event (II) for pure SBA-15 samples occurs from 180 to 1000 °C, with approximately 4% weight loss attributed to the condensation of silanol groups on the SBA-15 surface, accompanied by an exothermic peak in the DSC curves (Table 5 and Figure 5). For the SBA-15:dANA composites, the second weight-loss event (II) occurs from 180 to 650 °C, with around 5% weight loss due to the condensation of the residual silanol groups and the dANA. The third weight-loss event occurs from 650 to 1000 °C, with a weight loss ranging from 40% to 43%, corresponding to the carbonaceous materials originating from the degradation of dANA and of alkali metals (KCl and NaCl) from phosphate-buffered saline.^19,31^

In the DSC curves (Figure 5) of the SBA-15:dANA composites, a distinct exothermic peak is observed at around 700 °C, characteristic of the melting point of the alkali metal salts (KCl and NaCl) present in the PBS buffer. This event was also reported by Miranda et al.^31^ in the DSC analysis of SBA-15 samples incorporated with BSA using PBS as the incorporation medium and was observed in the analysis of the pure salts.

The TG/DTG curves of dANA in PBS (evaporated for analysis) are shown in Figure S4. The first weight-loss event (10%) from 25 to 180 °C corresponds to the elimination of water molecules. The second weight-loss event (180–540 °C) is attributed to the decomposition of dANA (71%), and the last weight-loss event (12.8%), above 540 °C, is due to the decomposition of PBS salts,^19^ along with the elimination of residual carbonaceous materials.

The decomposition of pure PBS salts from 650 to 1000 °C results in an 82% weight loss.^19,31^ Considering this percentage and the composite mass ratio of SBA-15:dANA composites (10SBA–15:1dANA:8.7PBS), the nominal mass percentage of PBS salts in this temperature range (650–1000 °C) in the SBA-15:dANA composites is 36%. The total weight loss of the second and third weight loss events (around 45 to 48%) aligns with the nominal values of silanol groups (around 4%), dANA (5%), and PBS salts (36%), corroborating the TGA data and nominal composite mass ratios.

The absorption spectra in the infrared region of the pure SBA-15 samples (S1, S2, S3, and S4) and those incorporated with dANA (S1dANA, S2dANA, S3dANA, and S4dANA) are shown in Figure 6. After extraction with ethanol and calcination at 550 °C, none of the SBA-15 samples exhibited characteristic bands attributable to the template (Pluronic P123) used during their synthesis. Specifically, these bands are observed at 2970 and 2875 cm^–1^, correspond to the stretching vibrations of methyl (CH_3_) and methylene (CH_2_) groups, while those at 1465 and 1365 cm^–1^ are associated with the bending vibrations of CH_2_ and CH_3_ groups^31^ (Figure 5A,B). Additionally, stretching vibrations (νO–H) of silanol groups (Si–OH) and adsorbed water were observed at around 3381 cm^–1^, along with a bending vibration band of the O–H groups (δO–H) at 1640 cm^–1^. The band at 3743 cm^–1^ was attributed to the stretching vibration of the free-silanol groups (Si–OH) on the surface of silica. The broad peak observed between 1000 and 1250 cm^–1^ corresponds to the asymmetric stretching of siloxane groups (Si–O–Si).^43^ The band at 965 cm^–1^ was attributed to the asymmetric vibration of the Si–OH group, while the bands around 800 and 450 cm^–1^ were assigned to Si–O–Si symmetric stretching and out-of-plane deformations, respectively.^44^ In the FTIR spectra of all the SBA-15:dANA composites, the bands associated with pure SBA-15 were observed without significant shifts. However, the bands related to free-SiOH groups were absent, and those bands related to protein amides were present. The disappearance of the free-SiOH bands indicates interactions between silica and the protein. The incorporation of dANA into the SBA-15 matrix was confirmed by the presence of the amide I (1649 cm^–1^) and amide II (1521 cm^–1^) bands.^31^

**Figure 6:**
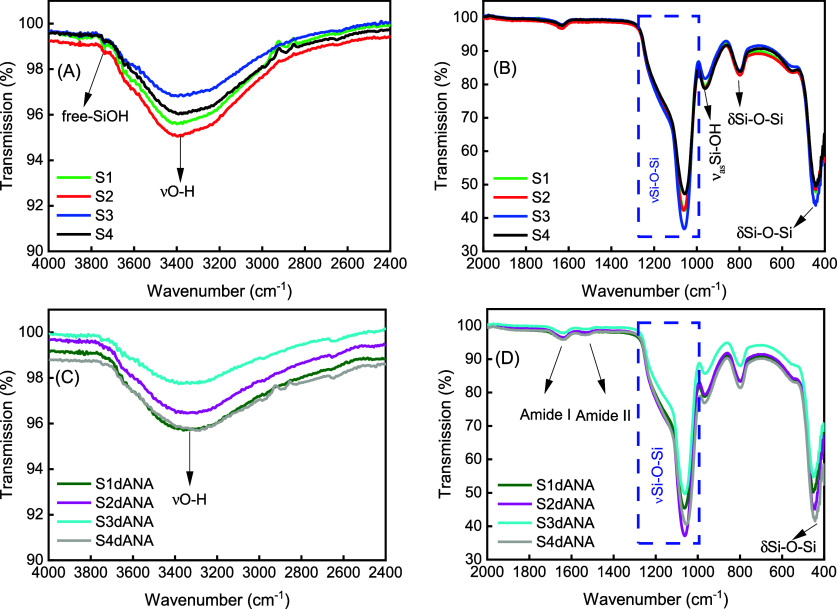
FTIR spectra of pure SBA-15 samples (S1, S2, S3, and S4) (A, B) and S1dANA, S2dANA, S3dANA, and S4dANA composites (C, D).

The characteristic vibration of free silanol groups (Si–OH), typically observed at around 3700 cm^–1^, was evident on the surface of the free silica particles. To further investigate silica-protein interactions, we monitored the vibration spectra of functional groups within the 4000–2400 cm^–1^ region for both the free silica particles and the protein-incorporated silica (composites). It is also known that the interaction of silanol groups in hydrogen bonding is known to cause broadening and often an apparent increase in intensity in the ∼3200 cm^–1^ region. Examination of the IR spectra (4000–2400 cm^–1^ region) revealed a distinct change in the shape for the composites, which became more symmetrical. Further evidence of the interaction is provided by the disappearance or significant reduction of the intensity of the band at ∼3700 cm^–1^ (attributed to free silanols) in the spectra of the composites. Following protein incorporation, it is anticipated that not all available silanol groups on the silica surface will be engaged in interaction with the protein. Consequently, this free silanol band is expected to be less prominent, though potentially still detectable, in some composite samples, as is possibly the case of S2dANA and S3dANA.

The dANA sample in solution and the S1dANA, S2dANA, S3dANA, and S4dANA composites were analyzed by fluorescence spectroscopy with excitation performed at a wavelength of 280 nm (Figure 7). All these samples exhibited maximum emission centered at 320 nm, indicating that the aromatic microenvironment of tryptophan residues was preserved from exposure to solvent after the incorporation of dANA into the different samples. Except that, the emission spectrum of the S3 sample exhibited two substantial peaks at 356 and 372 nm. This shift to a longer wavelength (red shift) may indicate an alteration in the microenvironment surrounding the tryptophan residues in the protein, suggesting a greater exposure of the tryptophan residues to the polar solvent, which is consistent with the protein (dANA) being located within the mesopores and macropores of SBA-15 (S3).^45^ However, this change has not significantly affected the protein secondary structure (dANA), thus, preserving its antigenic function.

**Figure 7:**
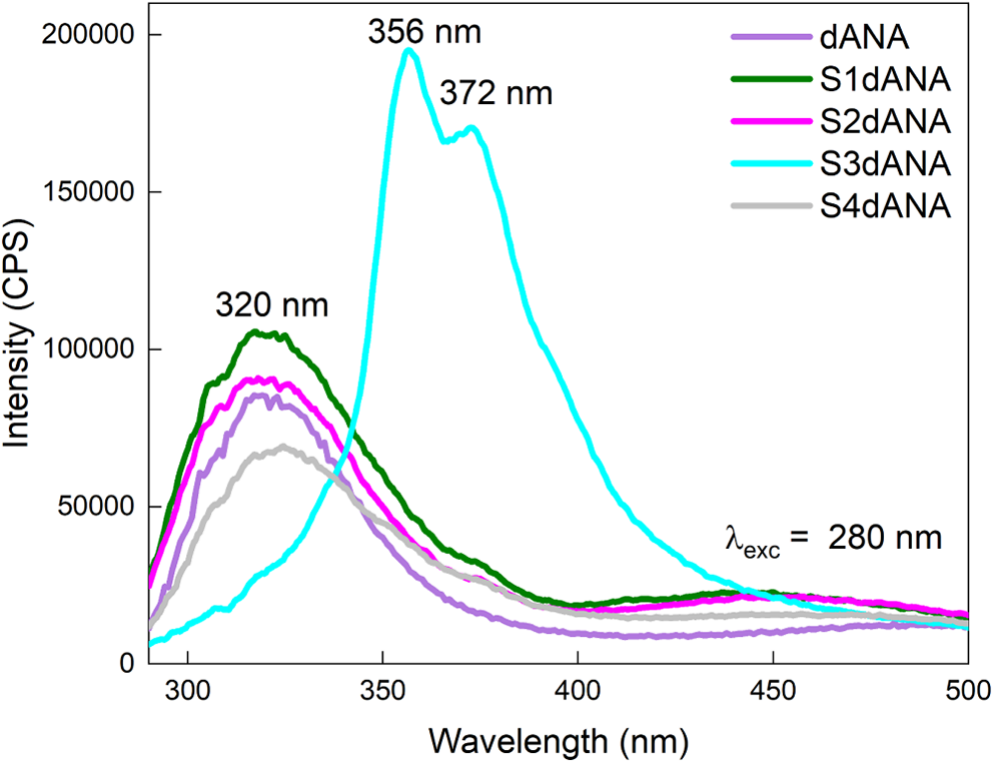
Fluorescence spectra of dANA in solution and SBA-15:dANA composites, excitation was performed at 280 nm.

Synchrotron radiation circular dichroism (SRCD) analyses were performed to track the protein’s secondary structure before and after protein incorporation into silica and to further investigate the fluorescence analysis of the S3dANA sample (whether the native secondary structure of the protein could have been lost). Figure 8 shows the SRCD spectra of dANA in solution and its composites. All the SRCD spectra of dANA exhibited two absorption minima at 220 and 208 nm and a maximum at 192 nm, which are characteristic of the α-helix content present in the native structure (34% helix).^19,31^

**Figure 8:**
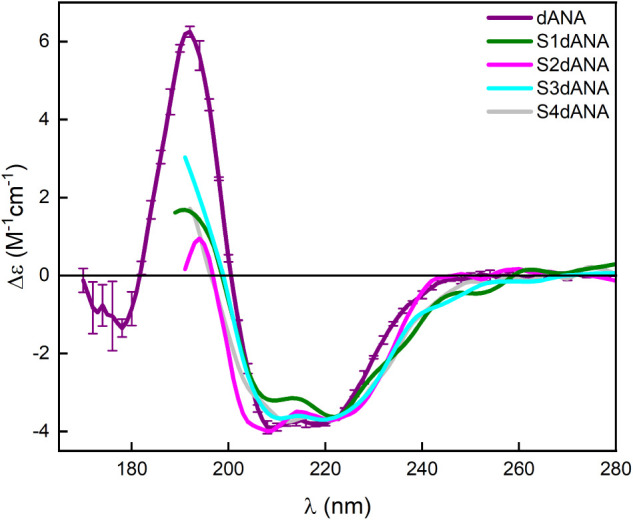
SRCD spectra of dANA in solution and SBA-15 incorporated with dANA.

Overall, in the composites’ SRCD spectra, the typical line shape and the spectral bands corresponding to the dANA native structure were preserved, suggesting that the structural content of the protein (34% helix and 21% ribbon-β) remained intact after incorporation into the SBA-15 particles. Small broadenings around the peaks at 208 and 222 nm are observed, which can be attributed to the increased scattering caused by the presence of the silica particles. These results indicate that all the SBA-15 samples incorporated with dANA, particularly S3dANA, have not caused any severe structural alteration or led to protein unfolding/denaturation but instead preserved the dANA native structure, consequently suggesting the preservation of its antigenic function. It is worth noting that the original structure of dANA was preserved in the S3dANA sample, which shows that there was no denaturation, but rather specific interactions around the tryptophan residues’ microenvironment.

SBA-15 is a promising material for use in vaccine adjuvant applications; therefore, evaluating the toxicity and biocompatibility of SBA-15:dANA composites is crucial before conducting *in vivo* immunogenicity assays. The hemolytic activity assay was selected as a straightforward and rapid method for initial toxicity and biocompatibility screening, using viable erythrocytes. This assay can provide preliminary indications of the toxicity for the various SBA-15:dANA composites and pure SBA-15 materials. While the literature suggests that SBA-15 incorporated with various antigens generally does not exhibit *in vivo* toxicity, the findings of Abbaraju et al.,^46^ prompted careful consideration. They demonstrated that silica nanoparticles with an asymmetric shape exhibit lower hemolytic activity (i.e., toxicity) compared to that of symmetrical nanoparticles. This phenomenon is attributed to the less aggressive and more localized interaction of asymmetric particles with the erythrocyte membrane, whereas symmetrical nanoparticles, having a larger contact area, can induce greater mechanical stress. This observation raised concerns that some of our SBA-15 morphologies could potentially induce toxicity. Consequently, we adopted the hemolytic activity test as an ethical screening strategy, adhering to the 3R principles (reduction, refinement, and replacement of animal testing), to preselect nontoxic samples, thereby aiming to minimize the number of animals required for subsequent *in vivo* studies. The hemolytic assays were performed using 100 μg of the SBA-15 component for both pure silica and the composites; the SBA-15:dANA contained dANA at 10% (w/w) relative to the SBA-15 mass. The SBA-15:dANA composites (S1dANA, S2dANA, S3dANA, and S4dANA) did not exhibit significant hemolytic effects. Hemolytic activity was found to be up to 1.5% for the pure SBA-15 samples (S1, S2, S3, and S4) and up to 2.15% for the SBA-15:dANA composites (S1dANA, S2dANA, S3dANA, and S4dANA). These values are well below the 5% threshold generally considered indicative of hemolytic concern, suggesting no significant damage to erythrocyte cell membranes (Figure 9). As shown in Figure 9, no red coloration was observed in the supernatant for any tested samples, in stark contrast to the 100% hemolysis control (Triton X-100 treated), which was distinctly red. This lack of coloration indicates that hemoglobin was not released and that the erythrocytes remained intact. These results suggest that all tested SBA-15 formulations are nonhemolytic under these *in vitro* conditions and, by this measure, preliminarily deemed nontoxic, rendering them suitable for the planned *in vivo* immunogenicity studies. Our findings align with other reports in the literature. For instance, Pędziwiatr-Werbicka et al.^47^ found that SBA-15 with hydrophobic surface modifications also did not exhibit significant hemolytic activity (toxicity). Furthermore, Ferenc et al.^48^ investigated the hemolytic potential of various silica types, including pure and functionalized (−NH_2_, −SH, −COOH) mesoporous SBA-15, similar to the materials in our study. They reported that even after 24 h of exposure to high concentrations (1000 μg mL^–1^), all their SBA-15 samples induced less than 5% hemolysis. This indicates that SBA-15 generally exhibits a low hemolytic toxicity even under stringent *in vitro* conditions, supporting its potential for *in vivo* applications.

**Figure 9:**
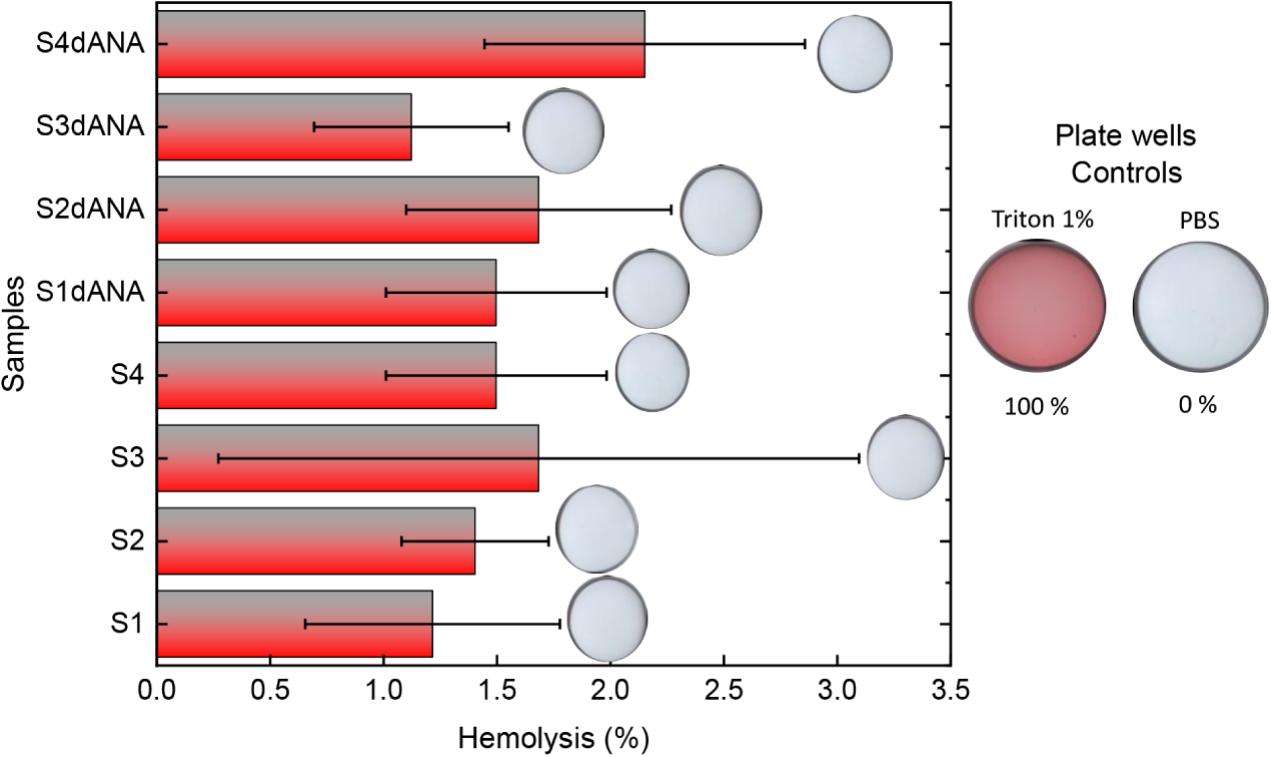
Hemolytic activity of pure SBA-15 samples (S1, S2, S3, and S4) and SBA-15:dANA composites (S1dANA, S2dANA, S3dANA, and S4dANA). Note: The circles at the top of the bars are the wells of the plate with the solutions tested, showing that the samples did not show a red color that would represent hemolysis and toxicity.

We studied the influence of different silica morphologies on the humoral response to dANA. The total IgG titers produced by HIII mice immunized with SBA-15 samples of different morphologies incorporated with dANA are shown in Figure 10 and Table 6. A significant difference was observed only in the primary response (30th day), where sample S3 showed significantly higher titers. This indicates that hexagonal morphology can expose the dANA to the antigen-presenting cells more quickly, resulting in a faster immune response compared to other morphologies (S1dANA, S2dANA, and S4dANA). However, the titers increased and equalized across all samples in the secondary response, indicating good secondary immunogenic responses from all morphologies.

**Figure 10:**
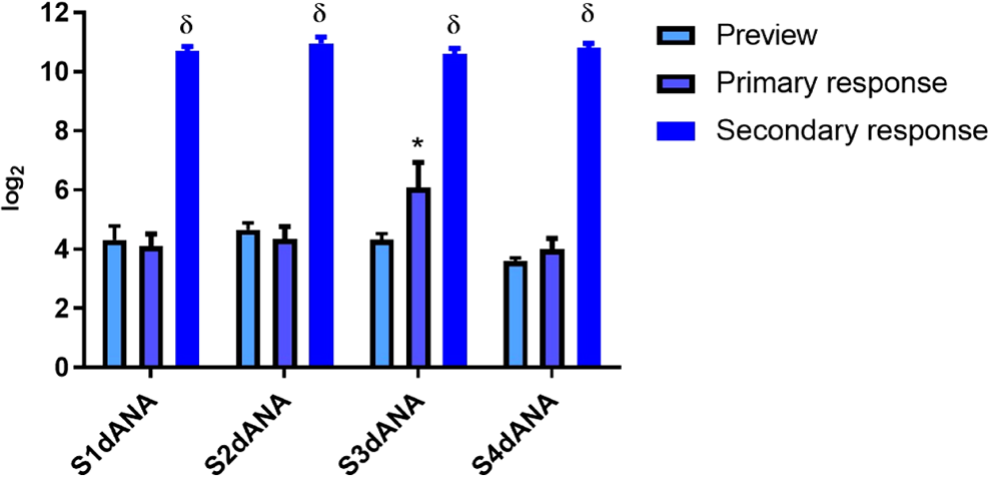
Anti-dANA (diphtheria anatoxin) antibody response (log_2_ titers) of male and female animals of the HIII strain immunized subcutaneously with SBA-15:dANA composites (S1dANA, S2dANA, S3dANA, and S4dANA). The results are presented as mean ± SD (*N* = 5). * visualization × primary response; δ secondary response × other groups. Considered significant at *p* < 0.01.

**Table 6:** Anti-dANA (Diphtheria Anatoxin) Antibody Response (log_2_ Titers) of Male and Female Animals of the HIII Strain Immunized Subcutaneously with SBA-15:dANA Composites (S1dANA, S2dANA, S3dANA, and S4dANA)

Samples	Preview (log_2_)	Primary response (log_2_)	Secondary response (log_2_)
S1dANA	4.30	4.11	10.70
S2dANA	4.66	4.64	10.96
S3dANA	4.26	5.73	10.58
S4dANA	3.60	3.99	10.81

Comparing these results with those of Rasmussen et al.,^19^ it can be seen that the SBA-15 samples with different morphologies incorporated with dANA tested in this study demonstrate better immunogenic activities than pure dANA when administered orally and incorporated into Al­(OH)_3_, which is the main adjuvant used in vaccine formulations. This was also confirmed by Trezena et al.,^18^ where the dANA and tANA (tetanus anatoxin) antigens were incorporated into SBA-15 showed a greater immunological response than the two antigens without the SBA-15-based adjuvant. These studies by Rasmussen et al.^19^ and Trezena et al.^18^ support the efficiency of SBA-15 as a vaccine adjuvant for diphtheria.

The greater immunological response observed in the S3dANA sample may be linked to the higher dANA load in the mesopores and the specific interaction between dANA and SBA-15, which could enhance the antigen dANA exposure to the immune system. However, since all SBA-15:dANA composites showed a satisfactory secondary immunological response, the different morphologies tested do not limit the immunogenic activity of the SBA-15:dANA composites.

The initial objective of this study was to evaluate the adjuvant/protective effect of SBA-15 on the final phenotype resulting from immunizations, specifically the quantitative production of antidiphtheria antibodies. We believe that the next phase of this work, building upon this initial evaluation, will involve a deeper investigation of the cellular and molecular mechanisms underlying the phenotypic conditions that we consider most effective.

The use of SBA-15 mesoporous silica as a model system in biomedical research has garnered significant interest due to its well-defined pore structure, high surface area, and tunable physicochemical properties. These characteristics render SBA-15 a valuable tool for investigating fundamental mechanisms related to loading cargo molecules, controlled release kinetics, and cellular uptake. Its ordered mesoporous architecture provides a highly reproducible and controllable platform for early-stage studies, enabling systematic evaluation of formulation parameters *in vitro*.^49^

Despite these advantages, the application of SBA-15 to actual vaccine systems presents inherent limitations, primarily because, as a model, it does not fully replicate the complexity of biological environments. Real-world vaccine carriers are typically designed to interact dynamically with the immune system and other physiological systems, features that SBA-15 lacks in its native form. For instance, they do not possess biological membranes, targeting ligands, or innate immunostimulatory components. Nevertheless, the nontoxic SBA-15 nanoparticles were shown to increase the immunogenicity and restore responsiveness in constitutively low-responder individuals, inducing both the IgG2a and IgG1 isotypes, independently of the immune cell commitment, thereby modulating the low-responder phenotype.^17^

Moreover, the SBA-15 surface chemistry, dominated by silanol groups, imparts a negative surface charge and lacks bioactive functionalities. However, chemical surface modification strategies can significantly improve its biological performance and transform SBA-15 into a more adaptable and biomimetic platform. These modifications allow researchers to emulate critical aspects of biologically derived systems while retaining the structural advantages of SBA-15.

A further consideration is the challenge of translating *in vitro* results obtained with SBA-15 to *in vivo* scenarios. While *in vitro* models are valuable for mechanistic insights and preliminary toxicity screening, they cannot fully reproduce the complexity of systemic biological processes such as enzymatic degradation, immune clearance, protein corona formation, and organ-specific biodistribution. Therefore, although SBA-15 serves as an effective antigen carrier, findings should be interpreted with caution and ideally complemented by data from more biologically relevant, biodegradable systems. Nonetheless, with appropriate surface engineering and complementary biological validation, SBA-15 remains a promising and versatile tool for the early phases of vaccine delivery research.

## 4 Conclusions

The modification of the synthesis conditions proved to be effective in producing different morphologies, including rope-shaped aggregated rods (S1), filiform rods (S2), hexagons, and nanospheres (S3 and S4). Despite the morphological differences, all samples retained their two-dimensional hexagonal mesoporous structure with only minor variations in lattice parameters. SAXS and USAXS modeling analyses further confirmed that samples S1, S2, and S3 exhibited a well-organized hexagonal structure with the incorporated material homogeneously distributed within the pores, forming large oblate ellipsoidal shapes. Additionally, the presence of globular clusters suggests structural consistency among these samples, supporting a synthesis approach that favors such organization. Sample S4, on the other hand, showed a biphasic structure, combining different mesopore arrangements and long prolate ellipsoids, indicating a more directed orientation of the incorporated material, more logically influenced either by the synthesis procedure, by specific interactions with the porous matrix, or because of the high aggregation level of the nanospheres typical of their nanometric sizes. The SAXS and NAI results suggest that, particularly in samples S1 and S4, the antigen was incorporated in both the mesopores and macropores of SBA-15. FTIR, TG/DSC, NAI, and fluorescence spectroscopy results show the presence of dANA in all samples. The differential interaction of tryptophan with the S3dANA sample indicated a greater exposure of tryptophan to the microenvironment within the mesopores of SBA-15. This suggests enhanced interaction with hydrophobic microenvironments due to the localization of dANA within both the meso- and macropores of SBA-15. SRCD analyses further confirmed the preservation of the native secondary structure of the protein in all of the SBA-15:dANA composites. Moreover, all samples exhibited no significant toxicity in the hemolytic assay. In the immunogenicity analysis, sample S3 demonstrated a significant increase in the primary immune response, possibly due to more effective antigen exposure to immune cells. In the secondary responses, all of the samples showed an efficient immunogenic response.

In conclusion, the synthesis techniques employed were efficient in producing SBA-15 particles with distinct morphologies, while preserving their mesoporous structure. Notably, these particles demonstrated excellent biocompatibility and elicited a robust immune response, underscoring their potential as promising vaccine adjuvants.

## Supplementary Material



## Notes

Detail on the modeling for sample S4 and S4dANA (Figure S1), IFT results for the USAXS data (Figure S2), *Ab initio* modeling for the USAXS data (Figure S3), TG/DSC curves of pure dANA (Figure S4), Obtained radius of gyration from the IFT fits (Table S1) (PDF)

## References

[1] Facciolà A., Visalli G., Laganà A., Di Pietro A. (2022). An Overview
of Vaccine Adjuvants: Current Evidence and Future Perspectives. Vaccines.

[2] World Health Organization. WHO Coronavirus (COVID-19) Dashboard, https://covid19.who.int/. (Accessed 10, 04, 2023).

[3] Polack F. P., Thomas S. J., Kitchin N., Absalon J., Gurtman A., Lockhart S., Perez J. L., Pérez Marc G., Moreira E. D., Zerbini C. (2020). Safety
and Efficacy
of the BNT162b2MRNA COVID-19 Vaccine. N. Engl.
J. Med..

[4] Ndwandwe D., Wiysonge C. S. (2021). COVID-19 Vaccines. Curr. Opin.
Immunol..

[5] Tan J., Ding B., Teng B., Ma P., Lin J. (2022). Understanding
Structure–Function Relationships of Nanoadjuvants for Enhanced
Cancer Vaccine Efficacy. Adv. Funct. Mater..

[6] Brito L. A., O’Hagan D. T. (2014). Designing
and Building the next Generation of Improved
Vaccine Adjuvants. J. Controlled Release.

[7] Nascimento I. P., Leite L. (2012). Recombinant Vaccines
and the Development of New Vaccine Strategies. Braz. J. Med. Biol. Res..

[8] Zhao T., Cai Y., Jiang Y., He X., Wei Y., Yu Y., Tian X. (2023). Vaccine Adjuvants:
Mechanisms and Platforms. Signal Transduct.
Target. Ther..

[9] Miranda M. C. R., Prezotti F. G., Borges F. A., Barros N. R., Cury B. S. F., Herculano R. D., Cilli E. M. (2017). Porosity Effects of Natural Latex
(Hevea Brasiliensis) on Release of Compounds for Biomedical Applications. J. Biomater. Sci., Polym. Ed..

[10] Wu Z., Liu K. (2021). Overview of Vaccine
Adjuvants. Med. Drug Discovery.

[11] Pasarin D., Ghizdareanu A.-I., Enascuta C. E., Matei C. B., Bilbie C., Paraschiv-Palada L., Veres P.-A. (2023). Coating Materials to Increase the
Stability of Liposomes. Polymers.

[12] Takamori D. Y., Bizeto M. A., de Abreu
Fantini M. C., Rubinger C. P. L., Faez R., Martins T. S. (2019). Polyaniline
Inclusion into Ordered Mesoporous Silica
Matrices: Synthesis, Characterization and Electrical Transport Mechanism. Microporous Mesoporous Mater..

[13] Cavalcante C. T., Molina C., Martins T. S. (2019). Synthesis
and Characterization of
Ordered Mesoporous Silica Containing Di-Ureasil Hybrid/Phosphotungstic
Acid and Eu3+. J. Mater. Sci.: Mater. Electron..

[14] Imperor-Clerc M., Davidson P., Davidson A. (2000). Existence
of a Microporous Corona
around the Mesopores of Silica-Based SBA-15 Materials Templated by
Triblock Copolymers. J. Am. Chem. Soc..

[15] Wan Y., Zhao D. (2007). On the Controllable
Soft-Templating Approach to Mesoporous Silicates. Chem. Rev..

[16] Jardim A. A., Bacani R., Goncalves N. S., Fantini M. C. A., Martins T. S. (2017). SBA-15:
TiO2 Nanocomposites: II. Direct and Post-Synthesis Using Acetylacetone. Microporous Mesoporous Mater..

[17] Carvalho L. V., Ruiz R. D. C., Scaramuzzi K., Marengo E. B., Matos J. R., Tambourgi D. V., Fantini M. C. A., Sant’anna O. A. (2010). Immunological
Parameters Related to the Adjuvant Effect of the Ordered Mesoporous
Silica SBA-15. Vaccine.

[18] Trezena A. G., Oseliero Filho P. L., da Silva L. C. C., Oliveira C. L. P., de
Souza Lopes J. L., da Silva Antonio N., Dettmann V. F. B., Akamatsu M. A., da Silva Martins T., Ribeiro O. G. (2022). Adjuvant Effect of Mesoporous
Silica SBA-15 on Anti-Diphtheria and Anti-Tetanus Humoral Immune Response. Biologicals.

[19] Rasmussen M. K., Bordallo H. N., Bordenalli M. A., Akamatsu M. A., Trezena A. G., Tino-De-Franco M., Sant’anna O. A., da Silva Martins T., de Souza Lopes J. L., de Abreu Fantini M. C. (2021). Assessing the Efficiency
of SBA-15 as a Nanocarrier for Diphtheria Anatoxin. Microporous Mesoporous Mater..

[20] Mechler-Dreibi M. L., Almeida H. M. S., Sonalio K., Martines M. A. C., Petri F. A. M., Zambotti B. B., Ferreira M. M., Storino G. Y., Martins T. S., Montassier H. J. (2021). Oral Vaccination of Piglets against Mycoplasma
Hyopneumoniae Using Silica SBA-15 as an Adjuvant Effectively Reduced
Consolidation Lung Lesions at Slaughter. Sci.
Rep..

[21] de
Pádua Oliveira D. C., de Barros A. L. B., Belardi R. M., de Goes A. M., de Oliveira Souza B. K., Soares D. C. F. (2016). Mesoporous Silica Nanoparticles as a Potential Vaccine
Adjuvant against Schistosoma Mansoni. J. Drug
Deliv. Sci. Technol..

[22] Mercuri L. P., Carvalho L. V., Lima F. A., Quayle C., Fantini M. C. A., Tanaka G. S., Cabrera W. H., Furtado M. F. D., Tambourgi D. V., Matos J. D. R. (2006). Ordered Mesoporous Silica SBA-15: A New Effective
Adjuvant to Induce Antibody Response. Small.

[23] Wang T., Jiang H., Zhao Q., Wang S., Zou M., Cheng G. (2012). Enhanced Mucosal and
Systemic Immune Responses Obtained by Porous
Silica Nanoparticles Used as an Oral Vaccine Adjuvant: Effect of Silica
Architecture on Immunological Properties. Int.
J. Pharm..

[24] Lee H. I., Kim J. H., Stucky G. D., Shi Y., Pak C., Kim J. M. (2010). Morphology-Selective Synthesis of Mesoporous SBA-15
Particles over Micrometer, Submicrometer and Nanometer Scales. J. Mater. Chem..

[25] Zhao D., Feng J., Huo Q., Melosh N., Fredrickson G. H., Chmelka B. F., Stucky G. D. (1998). Triblock
Copolymer Syntheses of Mesoporous
Silica with Periodic 50 to 300 Angstrom Pores. Science.

[26] Hammersley A. P. (2016). FIT2D:
A Multi-Purpose Data Reduction, Analysis and Visualization Program. J. Appl. Cryst..

[27] Complex Fluids Group (GFCx) at the Physics Institute in São Paulo University (IFUSP) EMUSAXS center-. https://portal.if.usp.br/emu/pt-br/node/323 (Accessed 03, 04, 2025).

[28] Oliveira, C. L. P. ; Vorup-Jensen, T. ; Andersen, C. B. F. ; Andersen, G. R. ; Pedersen, J. S. Discovering New Features of Protein Complexes Structures by Small-Angle X-Ray Scattering Applications of Synchrotron Light to Scattering and Diffraction in Materials and Life Sciences Springer Berlin, Heidelberg 2009 776 231–244 10.1007/978-3-540-95968-7_11

[29] Losito D. W., de Araujo D. R., Bezzon V. D. N., Oseliero Filho P. L., Fonseca F. L. A., Chagas C. D. S., Barbosa E., Oliveira C. L. P., Fantini M. C. D. A., Ferreira F. F. (2021). Mesoporous
Silica–Fe3O4 Nanoparticle Composites as Potential Drug Carriers. ACS Appl. Nano Mater..

[30] Miles A. J., Wallace B. A. (2018). CDtoolX, a Downloadable Software Package for Processing
and Analyses of Circular Dichroism Spectroscopic Data. Protein Sci..

[31] Miranda M. C. R., Nunes C. M., Santos L. F., da Silva L. B., de Jesus V. R., Andréo Filho N., Pedro J. A. F., Lopes J. L. S., Oliveira C. L. P., Fantini M. C. A. (2024). Ordered Mesoporous Silicas
for Potential Applications in Solid Vaccine Formulations. Vaccine.

[32] Onuma Y., Satake M., Ukena T., Roux J., Chanteau S., Rasolofonirina N., Ratsimaloto M., Naoki H., Yasumoto T. (1999). Identification
of Putative Palytoxin as the Cause of Clupeotoxism. Toxicon.

[33] Garcia P. R. D. A. F., Bicev R. N., Oliveira C. L. P., Sant’anna O. A., Fantini M. C. D. A. (2016). Protein Encapsulation in SBA-15 with Expanded Pores. Microporous Mesoporous Mater..

[34] Jardim, A. A. M. L. F. Nanocompósitos TiO2: SBA-15 e Suas Potenciais Aplicações Em Fotocatálise e Fotoproteção. 2015, https://repositorio.unifesp.br/handle/11600/47343.

[35] Fonseca R. L. M., Sampaio D., Mayrink T. F., Alcamand H. A., Palhares H. G., Nunes E. H. M., Houmard M. (2022). Influence
of the Alcoholic Solvent
and Gelation Temperature on the Structural and Water Vapor Adsorption
Properties of Silica Adsorbents Synthesized by Sol-Gel Method without
Catalyst. Microporous Mesoporous Mater..

[36] Legnoverde M. S., Basaldella E. I. (2016). Influence
of Particle Size on the Adsorption and Release
of Cephalexin Encapsulated in Mesoporous Silica SBA-15. Mater. Lett..

[37] Sundblom A., Oliveira C. L. P., Palmqvist A. E. C., Pedersen J. S. (2009). Modeling in Situ
Small-Angle X-Ray Scattering Measurements Following the Formation
of Mesostructured Silica. J. Phys. Chem. C.

[38] Semenyuk A.
V., Svergun D. I. (1991). GNOM–a
Program Package for Small-Angle Scattering
Data Processing. J. Appl. Cryst..

[39] Svergun D. I. (1999). Restoring
Low Resolution Structure of Biological Macromolecules from Solution
Scattering Using Simulated Annealing. Biophys.
J..

[40] Volkov V. V., Svergun D. I. (2003). Uniqueness of *Ab Initio* Shape Determination
in Small-Angle Scattering. J. Appl. Cryst..

[41] Thommes M., Kaneko K., Neimark A. V., Olivier J. P., Rodriguez-Reinoso F., Rouquerol J., Sing K. S. W. (2015). Physisorption of Gases, with Special
Reference to the Evaluation of Surface Area and Pore Size Distribution
(IUPAC Technical Report). Pure Appl. Chem..

[42] Kruk M., Jaroniec M., Sayari A. (1999). Relations between Pore Structure
Parameters and Their Implications for Characterization of MCM-41 Using
Gas Adsorption and X-Ray Diffraction. Chem.
Mater..

[43] Beretta B., Conti A., Fiocchi A., Gaiaschi A., Galli C. L., Giuffrida M. G., Ballabio C., Restani P. (2001). Antigenic Determinants
of Bovine Serum Albumin. Int. Arch Allergy Immunol..

[44] Ellerbrock R., Stein M., Schaller J. (2022). Comparing Amorphous Silica, Short-Range-Ordered
Silicates and Silicic Acid Species by FTIR. Sci. Rep..

[45] Vivian J. T., Callis P. R. (2001). Mechanisms of Tryptophan
Fluorescence Shifts in Proteins. Biophys. J..

[46] Abbaraju P. L., Meka A. K., Song H., Yang Y., Jambhrunkar M., Zhang J., Xu C., Yu M., Yu C. (2017). Asymmetric
Silica Nanoparticles with Tunable Head–Tail Structures Enhance
Hemocompatibility and Maturation of Immune Cells. J. Am. Chem. Soc..

[47] Pędziwiatr-Werbicka E., Miłowska K., Podlas M., Marcinkowska M., Ferenc M., Brahmi Y., Katir N., Majoral J., Felczak A., Boruszewska A. (2014). Oleochemical-tethered
SBA-15-type Silicates with Tunable Nanoscopic Order, Carboxylic Surface,
and Hydrophobic Framework: Cellular Toxicity, Hemolysis, and Antibacterial
Activity. Chem. - Eur. J..

[48] Ferenc M., Katir N., Miłowska K., Bousmina M., Majoral J.-P., Bryszewska M., El Kadib A. (2015). Haemolytic Activity and Cellular
Toxicity of SBA-15-Type Silicas: Elucidating the Role of the Mesostructure,
Surface Functionality and Linker Length. J.
Mater. Chem. B.

[49] Scaramuzzi K., Tanaka G. D., Neto F. M., Garcia P. R. A. F., Gabrili J. J. M., Oliveira D. C. A., Tambourgi D. V., Mussalem J. S., Paixão-Cavalcante D., Orlando M. T. (2016). Nanostructured
SBA-15 Silica: An Effective Protective Vehicle to Oral Hepatitis B
Vaccine Immunization. Nanomedicine.

