# Supplementary material for: Adjuvant Effect of Mesoporous Silica SBA-15 of Different Morphologies on Antidiphtheria Immune Response
Source: ACS Omega. 2025 Jun 19;10(25):27534–49. doi: 10.1021/acsomega.5c03459 (PMC12223812; doi:10.1021/acsomega.5c03459)
Supplement: Supplementary file 1 [file ao5c03459_si_001.pdf]

Supporting Information

Adjuvant effect of mesoporous silica SBA-15 of different morphologies on anti-diphtheria immune response

Matheus C. R. Miranda[a], Carmen M. Nunes[a], Danilo W. Losito[a], Fernanda M. Rocha[a], Jéssica A. F. Pedro[b], Bruna C. Favoretto[d] , Gabriel B. M. Teobaldo[b], Luís C. C. da Silva[b], Jose L. S. Lopes[c], Cristiano L. P. Oliveira[b], Marcia C. A. Fantini[b], Orlando G. Ribeiro[d], Osvaldo A. Sant´Anna[d], Tereza S. Martins[a*]

[a]Instituto de Ciências Ambientais, Químicas e Farmacêuticas, Universidade Federal de São Paulo, Diadema, SP, Brazil.

[b]Instituto de Física, Universidade de São Paulo, São Paulo – SP, Brazil.

[c]Departamento de Física, Faculdade de Filosofia, Ciências e Letras de Ribeirão Preto, Universidade de São Paulo, Ribeirão Preto – SP, Brazil.

[d]Instituto Butantan, São Paulo – SP, Brazil.

*Corresponding authors:

Prof. Dr. Tereza S. Martins: tsmartins@unifesp.br

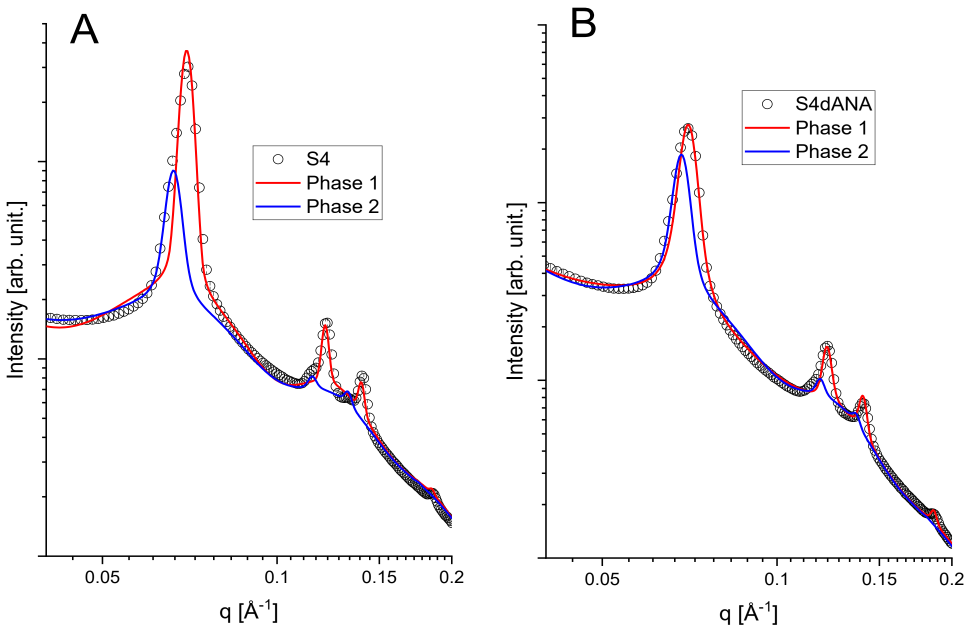

Figure S1 Detail on the modeling for sample S4 (A) and S4dANA (B) Symbols: experimental data. Solid lines: model fits. Red: phase 1, blue: phase 2. These samples have two phases (double peaks) and they were described it in the modeling.

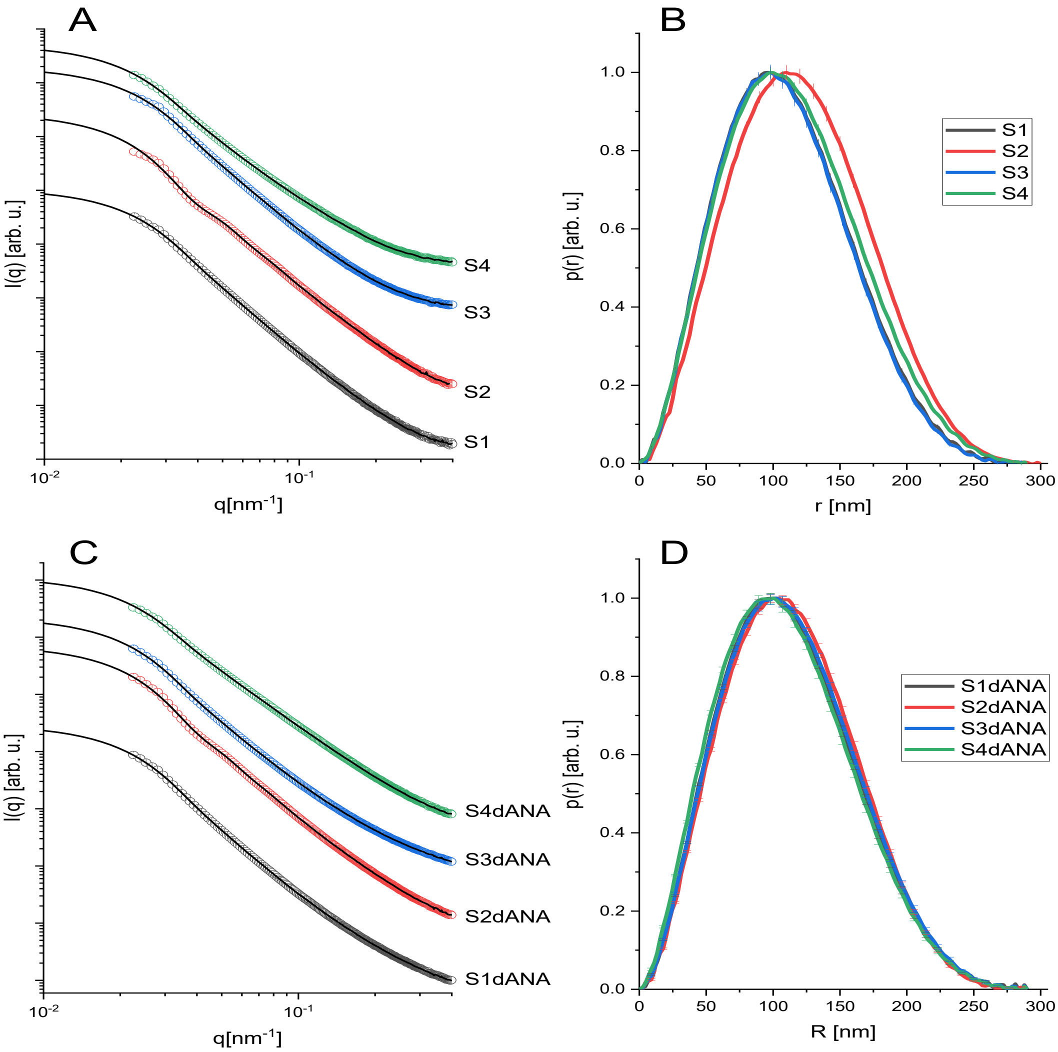

Figure S2 IFT results for the USAXS data. (A,C) Symbols: experimental data. Solid lines: IFT fits. (B,D) p(r) for the IFT fits.

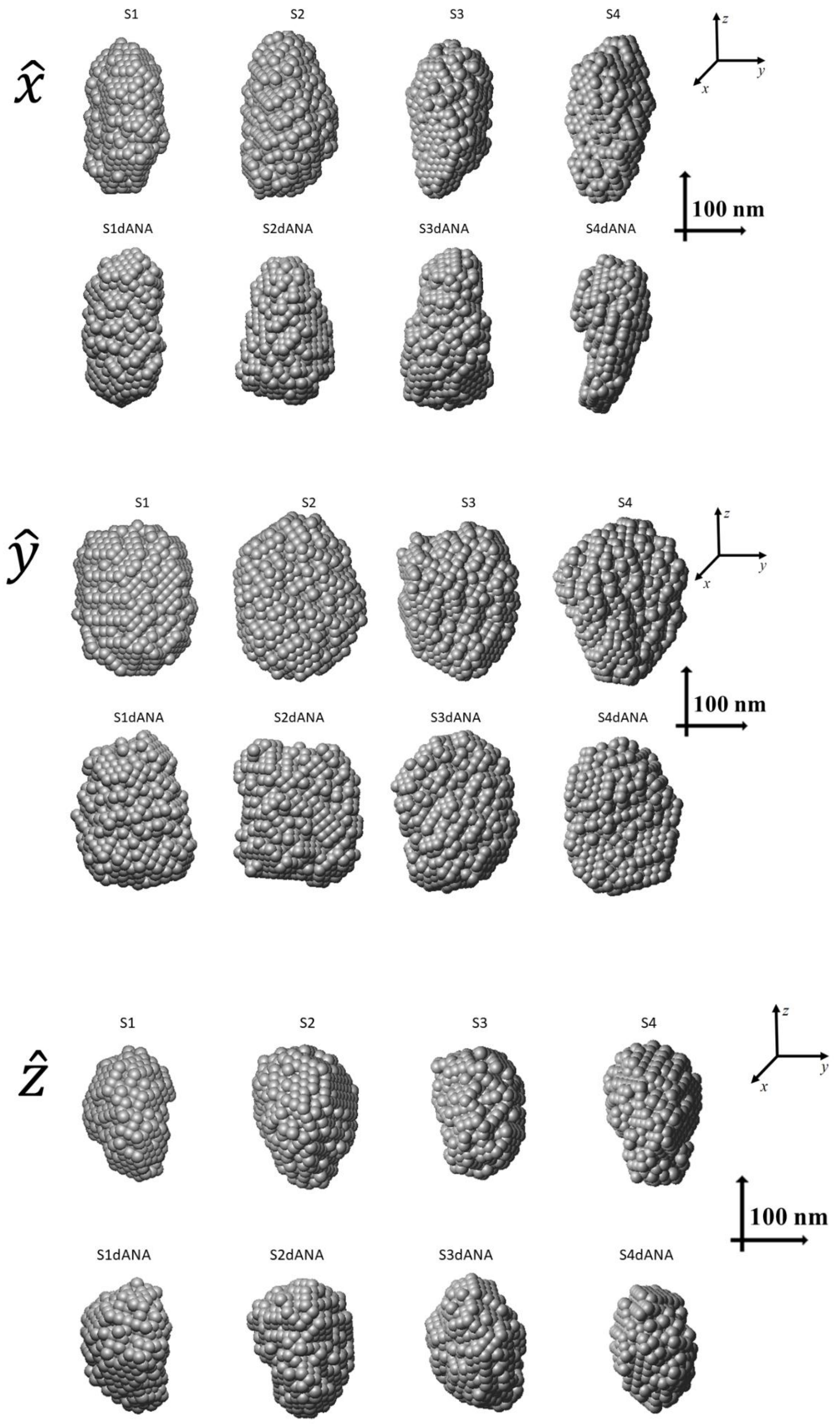

Figure S3 *Ab initio* modeling for the USAXS data. The three views along the x, y and z axis are shown.

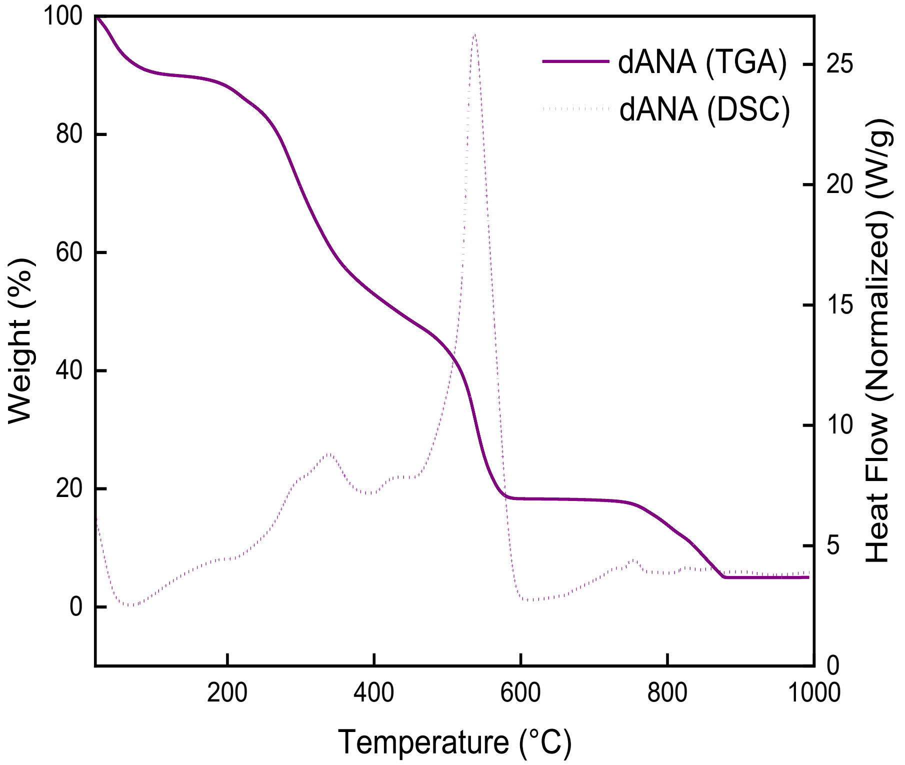

Figure S4 TG/DSC curves of pure dANA.

Table S1 Obtained radius of gyration from the IFT fits.

**Sample**	**RG [nm]**
**S1**	84.7±0.5
**S2**	91.9±0.3
**S3**	84.2±0.5
**S4**	87.7±0.4
**S1dANA**	85.7±0.4
**S2dANA**	86.8±0.3
**S3dANA**	86.7±0.4
**S4dANA**	84.6±0.5
